# White spot syndrome virus directly activates mTORC1 signaling to facilitate its replication via polymeric immunoglobulin receptor-mediated infection in shrimp

**DOI:** 10.1371/journal.ppat.1010808

**Published:** 2022-09-06

**Authors:** Pan-Pan Hong, Cang Li, Guo-Juan Niu, Xiao-Fan Zhao, Jin-Xing Wang

**Affiliations:** 1 Shandong Provincial Key Laboratory of Animal Cells and Developmental Biology, School of Life Sciences, Shandong University, Qingdao, Shandong, China; 2 State Key Laboratory of Microbial Technology, Shandong University, Qingdao, Shandong, China; Department of Biotechnology and Bioindustry Sciences, TAIWAN

## Abstract

Previous studies have shown that the mechanistic target of rapamycin complex 1 (mTORC1) signaling pathway has antiviral functions or is beneficial for viral replication, however, the detail mechanisms by which mTORC1 enhances viral infection remain unclear. Here, we found that proliferation of white spot syndrome virus (WSSV) was decreased after knockdown of *mTor* (mechanistic target of rapamycin) or injection inhibitor of mTORC1, rapamycin, in *Marsupenaeus japonicus*, which suggests that mTORC1 is utilized by WSSV for its replication in shrimp. Mechanistically, WSSV infects shrimp by binding to its receptor, polymeric immunoglobulin receptor (pIgR), and induces the interaction of its intracellular domain with Calmodulin. Calmodulin then promotes the activation of protein kinase B (AKT) by interaction with the pleckstrin homology (PH) domain of AKT. Activated AKT phosphorylates mTOR and results in the activation of the mTORC1 signaling pathway to promote its downstream effectors, ribosomal protein S6 kinase (S6Ks), for viral protein translation. Moreover, mTORC1 also phosphorylates eukaryotic translation initiation factor 4E-binding protein 1 (4EBP1), which will result in the separation of 4EBP1 from eukaryotic translation initiation factor 4E (eIF4E) for the translation of viral proteins in shrimp. Our data revealed a novel pathway for WSSV proliferation in shrimp and indicated that mTORC1 may represent a potential clinical target for WSSV control in shrimp aquaculture.

## Author summary

White spot syndrome virus (WSSV) is the causative pathogen of white spot disease (WSD) and represents the most destructive viral disease of shrimp. The virus has evolved various strategies to escape from host defenses or exploit host biological pathways for its reproduction. Studies on viral immune-escape mechanisms can provide new strategies for disease prevention and control in shrimp aquaculture. Mechanistic target of rapamycin (mTOR) plays a central role in the regulation of cell growth and metabolism, which nucleates two distinct protein complexes, mTOR complex 1 (mTORC1) and mTOR complex 2 (mTORC2) with diverse functions at different levels of the signaling pathway. mTORC1 is reported to be exploited by viruses in their reproduction. However, the detail mechanism remains unclear. In this study, we identified a new mechanism of mTOR being hijacked by WSSV in shrimp (*Marsupenaeus japonicus*). WSSV infects shrimp by its receptor, pIgR and induces the interaction of the intracellular domain of pIgR with Calmodulin. Calmodulin subsequently promotes the activation of AKT by interaction with the pleckstrin homology domain of the kinase. Activated AKT phosphorylates mTOR and results in the activation of the mTORC1 signaling pathway to promote its downstream effectors, S6Ks, for viral protein synthesis. Moreover, mTORC1 also phosphorylates 4EBP1, which results in the separation of 4EBP1 from eIF4E for the translation of viral proteins in shrimp. Our study reveals a novel strategy for WSSV proliferation in shrimp and indicates that the components of mTORC1 may represent potential clinical targets for WSSV control in shrimp aquaculture.

## Introduction

Mechanistic target of rapamycin (mTOR, also known as mammalian target of rapamycin) is a specific target protein of the drug, rapamycin, in eukaryotes [1]. Two mTOR homologs were originally identified in yeast [2], TOR1 and TOR2, whereas only one mTOR has been found to exist in other eukaryotes. mTOR belongs to the phosphatidylinositol-3 kinases (PI3K)-related kinase (PIKK) family and is an evolutionarily conserved serine/threonine protein kinase [3]. As an extremely important regulatory center in a variety of signaling pathways [4], mTOR controls several key biological processes, from protein synthesis to autophagy [5], mTOR has also manifold functions in regulation of innate immune responses in mammal [6].

mTOR nucleates two distinct protein complexes, mTOR complex 1 (mTORC1) and mTOR complex 2 (mTORC2), which contain both unique and shared components. The two complexes differ regarding their upstream pathways, downstream targets, as well as in their structures and functions [7–8]. Moreover, mTORC1 regulates both the synthesis of a series of biological molecules (e.g., proteins, lipids, and nucleotides) [9], and strongly inhibits autophagy [10]. Thus, the role of mTORC1 in vivo is to simultaneously promote anabolic metabolism and inhibit catabolic metabolism [11], with its main function to promote cell growth. mTORC1 mainly regulates protein translation through its downstream key effectors, ribosomal protein S6 kinase (S6Ks) [12] and eukaryotic initiation factor 4E binding protein (4EBPs) [13–14] to control protein synthesis. Compared with mTORC1, the function of mTORC2 is less well studied. It has been shown that mTORC2 senses the stimulation of growth factors and plays a role in cell survival and actin reorganization [15–16].

Several studies have demonstrated that a variety of viruses are capable of activating, reducing, or suppressing the mTOR signaling pathway to support their own replication in hosts [17]. In addition, a number of viruses prefer to attack upstream targets of mTORC1, such as PI3K (Phosphoinositide 3-kinase) or AKT (protein kinase B), for viral replication (e.g., Epstein-Barr virus [EBV] [18] and human papillomaviruses [HPV] [19]), whereas other viruses prefer to act on downstream targets by promoting 4EBP1 and S6K1 phosphorylation. For example, the non-structural protein 5A (NS5A) of Hepatitis C virus binds to the mRNA cap-binding eukaryotic translation initiation 4F (eIF4F) complex and up-regulates host translation initiation machinery through phosphorylation 4EBP1 for translation of a select group of proteins beneficial to HCV infection [20]. While the majority of viruses hijack the mTOR signaling pathway, a subset of viruses can be inhibited by the mTOR pathway, such as the mosquito-transmitted bunyavirus, Rift Valley fever virus (RVFV). RVFV infection can induce translational arrest through the eukaryotic initiation factor 4E binding protein 1/2 (4EBP1/2) (target molecules of mTORC1)-dependent decay of 5’-terminal oligopyrimidine mRNAs and result in the restriction of viral infection [21]. A recent study found that mTOR plays an essential role via the positive regulation of RIG I-like receptor-mediated antiviral function in human dendritic cells [22].

White spot syndrome virus (WSSV) is the causative agent of white spot syndrome and represents the most destructive viral disease, responsible for substantial economic loss, a total of more than $7 billion in the shrimp industry [23]. Understanding the mechanisms of WSSV infection is of great importance for the prevention and control of the disease in shrimp. Previous studies have found that WSSV can utilize different receptors or attachment proteins for its infection in shrimp [24–25]. Among these, only two of the reported receptors are genuine transmembrane proteins: 1) beta-integrins [26]; and 2) polymeric immunoglobulin receptor-like protein (pIgR) [27]. As a WSSV receptor, pIgR in shrimp contains a signal peptide, an extracellular domain including an IG domain and two IG-like domains, a transmembrane region and an intracellular region. It mediates viral endocytosis via the pIgR-CaM-Catherin pathway [27].

mTOR signaling pathway is at the center of multiple signaling pathways. Indeed, previous studies have shown that following WSSV infection in shrimp, the PI3K-Akt-mTOR-HIF1α pathway is activated and promotes fatty acid synthesis and lipid metabolism, ultimately promoting viral replication in the shrimp host [28]. In addition, during WSSV infection, the mTOR signaling pathway was also activated to promote glycolysis via the PI3K-Akt-mTOR pathway and enhance viral replication in shrimp [29]. However, the mechanism by which mTOR signaling is directly activated by different viruses for their protein synthesis in invertebrates remains unclear. In the present study, we identified a mTOR in *M*. *japonicus*. By knocking down *mTor* or an injection of rapamycin, the inhibitor of mTORC1, we found that mTORC1 signaling provided a beneficial role to WSSV proliferation. We further elucidated that WSSV infection directly activated mTORC1 signaling via the VP24-pIgR-CaM-AKT signaling cascade.

## Results

### WSSV exploits *mTor* to facilitate its replication in shrimp

The sequence, domain architecture, and phylogenetic analysis indicated that mTOR is a highly conserved protein in species ranging from shrimp to mammals (S1–S3 Figs). In addition, *mTor* is expressed in various tissues in shrimp, including hemocytes, heart, hepatopancrea, gills, stomach, and intestines in healthy shrimp (S4A Fig). We then investigated the *mTor* expression patterns at the mRNA level in shrimp challenged by WSSV. The results showed that *mTor* was significantly upregulated in the hemocytes (S4B Fig), gills (S4C Fig), and intestines (S4D Fig) of the WSSV-infected shrimp.

**Fig 1:**
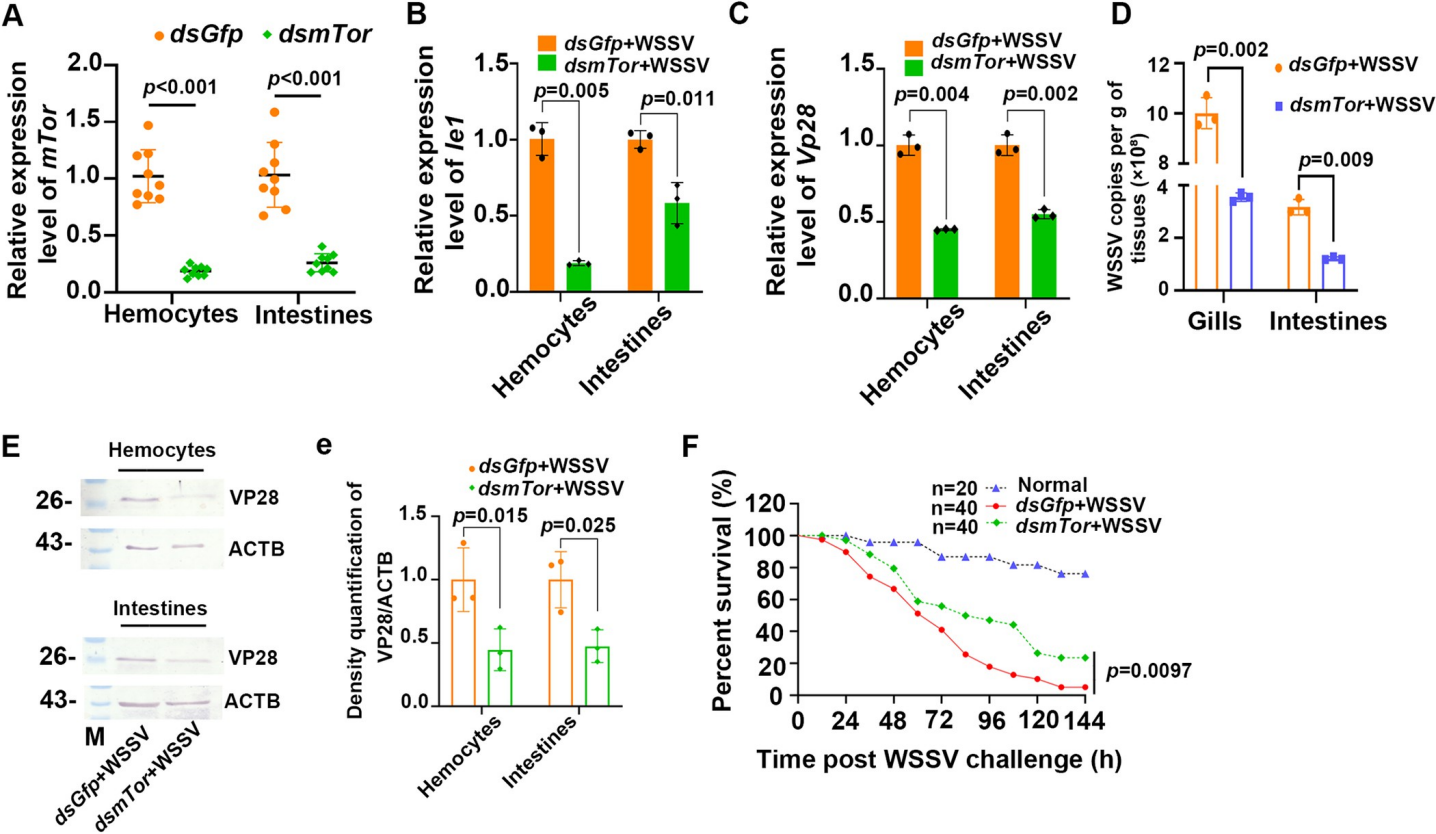
WSSV replication was suppressed and the survival rate increased following a knockdown of *mTor* in shrimp. (**A**) The efficiency of *mTor*-RNAi in hemocytes and intestines was detected by qPCR at 36 h post dsRNA injection. (**B**) *Ie1* expression in the hemocytes and intestines of *mTor*-RNAi shrimp following WSSV infection analyzed by qPCR at 36 h post WSSV injection. (**C**) *Vp28* expression in the hemocytes and intestines of *mTor*-RNAi shrimp challenged with WSSV at 36 h post WSSV injection. (**D**) The WSSV copy number in the gills and intestines of *mTor*-knockdown and control shrimp. (**E**) The level of VP28 protein expression in the hemocytes and intestines of *mTor*-RNAi shrimp challenged with WSSV and detected by Western blot at 36 hpi. ACTB (*β*-actin) was used as the loading control. (**e**) Statistical analysis of three independent experiments for panel E. Genomic DNA was extracted from the tissues of *mTor*-knockdown shrimp infected with WSSV at 36 hpi. Significant differences were analyzed using a Student’s *t*-test, *P* < 0.05 was considered to indicate a significant difference. (**F**) Survival rate of *mTor*-RNAi shrimp infected with WSSV. *dsGfp* injection was used as a control. The survival rate of each group was calculated, and the survival curves were presented as Kaplan-Meier plots. Differences between the two groups were statistically analyzed using a log-rank test in GraphPad Prism 8.0.

To explore the function of *mTor* in shrimp infected with WSSV, *mTor* RNA interference (RNAi) was performed and WSSV replication was analyzed using the expression of *Vp28* (the envelope protein gene of WSSV) and *Ie1* (the immediate early gene of WSSV) as indicators. Following the knockdown of *mTor* in the hemocytes and intestines of shrimp infected with WSSV (Fig 1A), the levels of *Ie1* and *Vp28* mRNA expression were significantly decreased in these tissues (Fig 1B and 1C). The level of VP28 protein expression was also decreased in the shrimp compared with the shrimp injected with *dsGfp* (Fig 1E and its statistical analysis 1e). Together, these results suggested that WSSV replication was significantly declined in the *mTor-*RNAi shrimp, which was also confirmed by the detection of WSSV copy numbers (Fig 1D). We further analyzed the survival rate of the shrimp, and the results showed that the survival rate of *mTor*-RNAi group significantly increased following WSSV infection compared with the *dsGfp*-injected group (Fig 1F). These results indicate that *mTor* is utilized by WSSV to facilitate its proliferation in shrimp.

### mTORC1 signaling was activated by enhancing 4EBP1 phosphorylation in response to WSSV infection in shrimp

**Fig 2:**
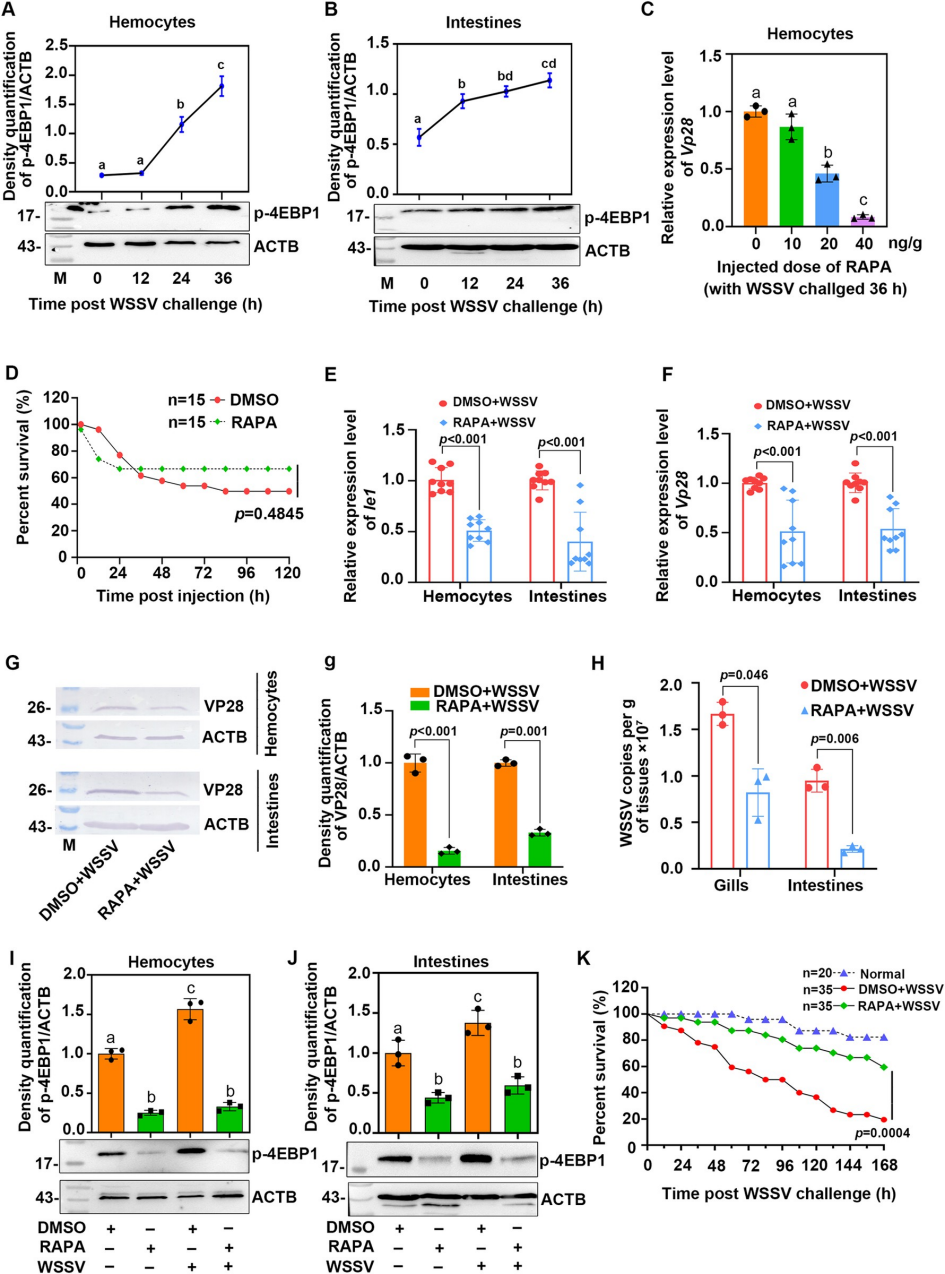
WSSV infection activates mTORC1 signaling and enhances WSSV proliferation. (**A** and **B**) The phosphorylation of 4EBP1 at different time points in the hemocytes (A) and intestines (B) of shrimp challenged with WSSV was analyzed by Western blot. The upper panel represents the statistical analysis of three independent experiments of the lower panel. (**C**) The effect of different doses of rapamycin on WSSV replication was analyzed using *Vp28* expression as an indicator. (**D**) The toxicity of rapamycin in shrimp was detected using a shrimp viability analysis following a rapamycin injection (20 ng/g body weight). (**E**) *Ie1* expression in the hemocytes and intestines of shrimp following an injection with rapamycin (20 ng/g body weight) analyzed by qPCR at 36 h post WSSV injection. (**F**) The level of *Vp28* expression in the hemocytes and intestines of shrimp following rapamycin injection (20 ng/g body weight) analyzed by qPCR at 36 hpi. (**G**) The level of VP28 protein expression in the hemocytes and intestines of shrimp following an injection with rapamycin in shrimp; (**g**) Statistical analysis of the three independent experiments in panel G. (**H**) WSSV copy numbers in the gills and intestines of shrimp injected with rapamycin followed by WSSV infection. (**I-J**) Changes in the level of 4EBP1 phosphorylation in hemocyes (I) and intestines (J) of shrimp following treatment with rapamycin and WSSV infection. The upper panel represents the statistical analysis of the lower panel. The mRNA, protein and genomic DNA used for WSSV replication analysis were extracted from hemocytes and different tissues of the shrimp infected with WSSV at 36 hpi. (**K**) The survival rate of rapamycin-injection shrimp following WSSV infection compared with the control group. The survival rate of each group was calculated, and the survival curves were presented as Kaplan-Meier plots. Differences between the two groups were statistically analyzed using a log-rank test in GraphPad Prism 8.0. Significant differences were analyzed using a Student’s *t*-test, *P* < 0.05 was considered to indicate a significant difference; different lowercase letters indicate significant differences (*P* < 0.05) in the one-way ANOVA analysis.

mTOR functions via the multiprotein complexes, mTORC1 and mTORC2. To understand the effect of mTOR on the proliferation of WSSV via mTORC1 or mTORC2 signaling in shrimp, we first detected the phosphorylation of the 4EBP1, the target protein of mTORC1, in the hemocytes and intestines of shrimp following WSSV infection. The results revealed that 4EBP1 phosphorylation were significantly increased in the hemocytes and intestines of the shrimp infected by WSSV (Fig 2A and 2B), indicating that mTORC1 was activated in the WSSV-infected shrimp. Rapamycin, the inhibitor of mTORC1, was injected into the shrimp following WSSV infection, and *Vp28* expression was analyzed as the indicator of the WSSV proliferation. The shrimp were injected with different doses of rapamycin, and the results showed that *Vp28* expression was significantly decreased in a dose-dependent manner in the shrimp (Fig 2C). Then the toxicity of rapamycin in the shrimp was further analyzed, the results showed that an injection with 20 ng/g rapamycin had no significant effect on shrimp mortality compared with the control group (Fig 2D). We selected an injection of 20 ng/g rapamycin for all subsequent experiments. The results showed that the level of *Ie1* and *Vp28* expression were both decreased substantially in the rapamycin-injection group compared with the DMSO-injection group following WSSV infection (Fig 2E and 2F). The same results were obtained with a VP28 protein expression analysis (Fig 2G and 2g). The WSSV copy number was also markedly decreased in the gills and intestines of rapamycin-injection shrimp infected with WSSV (Fig 2H). Furthermore, phosphorylation of 4EBP1 was significantly reduced in the shrimp injected with rapamycin following WSSV infection (Fig 2I and 2J). To further confirm that p-4EBP1 is regulated by mTORC1 in shrimp, we knocked down the specific target proteins in the mTORC1 and mTORC2 complex, *Raptor* and *Rictor*, and detected the phosphorylation level of 4EBP1. The results showed that following interference with *Raptor* by RNAi, the level of 4EBP1 phosphorylation was significantly decreased (S5C and S5c Fig). Finally, the survival rate of shrimp injected with rapamycin following WSSV infection was also analyzed. The survival rate of the shrimp increased significantly comparing with control (Fig 2K). Collectively, the above results suggest that WSSV infection activates the mTORC1 signaling pathway to promote WSSV proliferation in shrimp.

### WSSV exploits mTORC1 signaling for its proliferation via promoting the expression and phosphorylation of downstream target S6Ks

**Fig 3:**
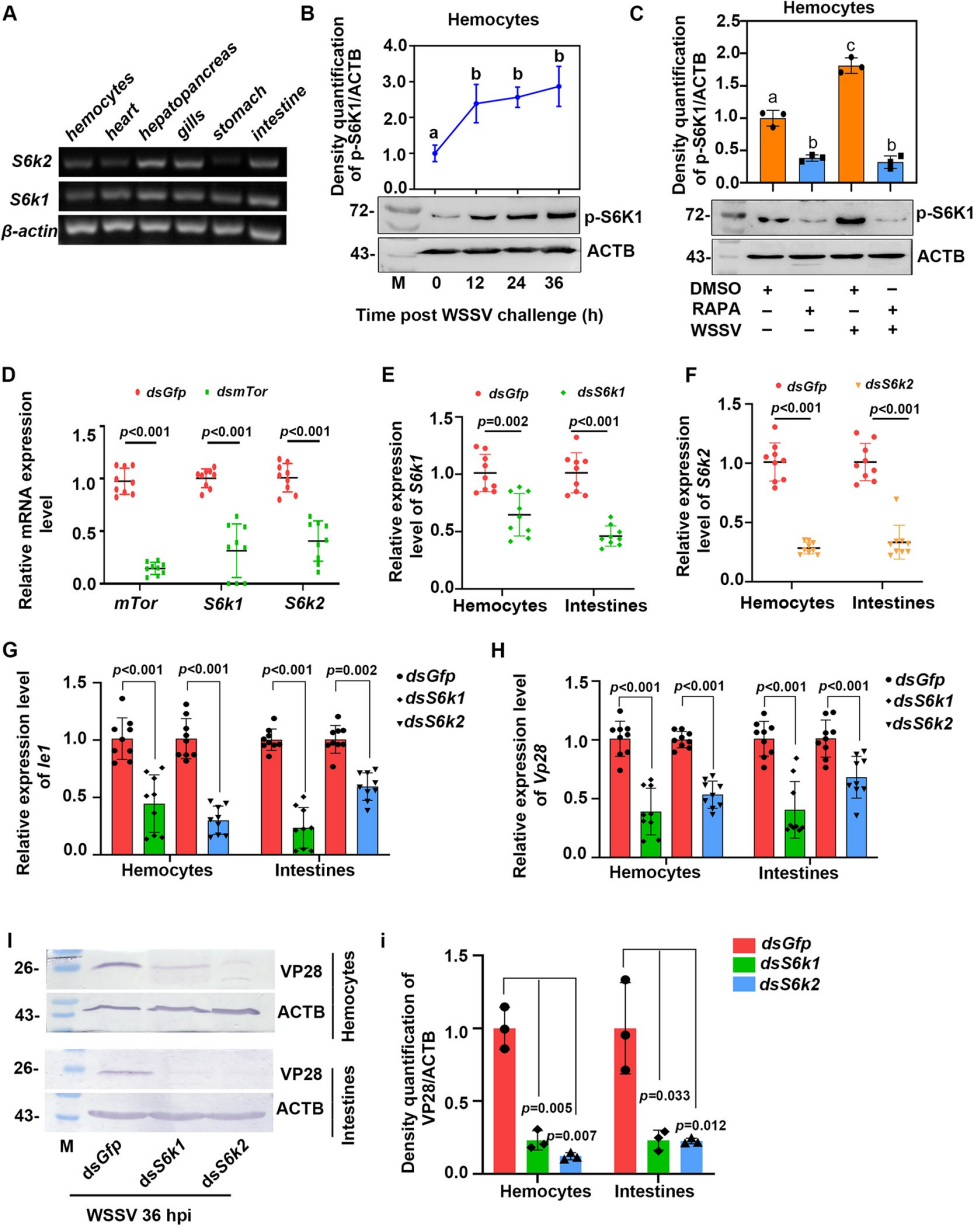
WSSV replication was suppressed in shrimp after a knockdown of downstream targets of mTORC1, *S6k1* and *dsS6k2*, following WSSV infection. (**A**) Tissue distribution of *S6k1* and *S6k2* in shrimp at the mRNA level detected using RT-PCR. (**B**) The phosphorylation of S6K1 at different time points in the hemocytes of shrimp challenged with WSSV analyzed by Western blot. The upper panel represents the statistical analysis of three independent experiments of the lower panel. (**C**) Changes in the level of S6K1 phosphorylation in hemocyes of shrimp following treatment with rapamycin and WSSV infection. In panel B and C, the subpanels of p-S6K1 and ACTB were from different gels with same amount of loading samples. (**D**) The mRNA expression level of *S6k1* and *S6k2* in the *mTor*-RNAi shrimp. (**E** and **F**) Efficiency of *S6k1-* (E) and *S6k2-*RNAi (F) in the hemocytes and intestines of shrimp as detected by qPCR. (**G** and **H**) The level of *Ie1* (G) and *Vp28* (H) mRNA expression in the hemocytes and intestines of shrimp following *S6k1* or *S6k2* interference. (**I**) The level of VP28 expression in the hemocytes and intestines following a knockdown of *S6k1* or *S6k2*. (**i**) Statistical analysis of three independent experiments for VP28 expression in shrimp. An injection with *dsGfp* was used as a control. The mRNA and protein used for WSSV replication analysis were extracted from hemocytes and intestines of the shrimp infected with WSSV at 36 hpi. Significant differences were analyzed using a Student’s *t*-test and significant differences were accepted at *P* < 0.05; different lowercase letters indicate significant differences (*P* < 0.05) in the one-way ANOVA analysis.

To verify the exploitation of mTORC1 signaling by WSSV for its infection, the expression of another downstream target in the signaling, ribosomal protein S6 kinases (S6K1 and S6K2 in S6A Fig), which are responsible for protein synthesis [30–31], were analyzed. We firstly detected the tissue distribution of *S6k1* and *S6k2*, and found that *S6k1* was distributed in all tested tissues, but *S6k2* was hardly detected in hemocytes and stomach (Fig 3A). Then the phosphorylation of S6K1 in shrimp challenged by WSSV was analyzed by western blot, the results showed that the phosphorylation of S6K1 increased significantly in the shrimp (Fig 3B), and phosphorylation of S6K1 was significantly inhibited in the shrimp injected with rapamycin both before and after WSSV infection (Fig 3C). After knockdown of *mTor*, the level of *S6k1* and *S6k2* expression declined substantially (Fig 3D). Next, *S6ks-*RNAi were performed to further explore the functions of *S6k1* and *S6k2* in WSSV-infected shrimp (Fig 3E and 3F), and results showed the levels of *Ie1* and *Vp28* mRNA were significantly decreased in the hemocytes and intestines of the shrimp compared with that of the *dsGfp*-injection group (Fig 3G and 3H). Moreover, VP28 expression was also analyzed at the protein level and the same results were obtained (Fig 3I and statistical analysis 3i). These results suggest that protein synthesis through mTORC1 signaling is utilized by WSSV through activation of S6K1 and S6K2 in shrimp.

### Inhibitor of AKT suppresses WSSV replication and 4EBP1 phosphorylation

**Fig 4:**
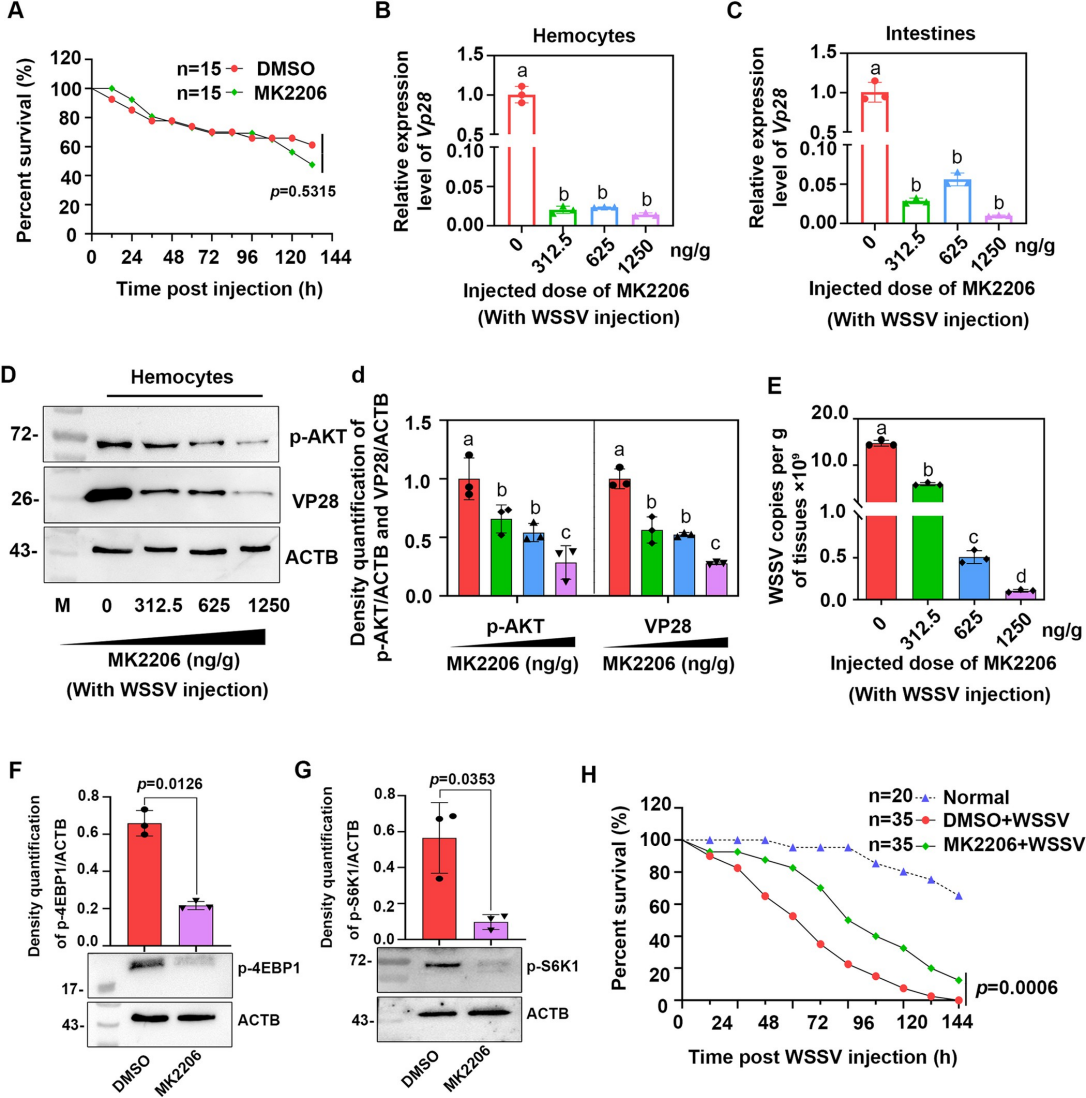
WSSV replication, 4EBP1 and S6K1 phosphorylation were inhibited by the AKT inhibitor, MK2206. (**A**) The toxicity of MK2206 on shrimp viability was detected following an injection with MK2206 (1250 ng/g body weight). The survival rate of each group was calculated, and the survival curves were presented as Kaplan-Meier plots. Differences between the two groups were statistically analyzed using a log-rank test in GraphPad Prism 8.0. (**B** and **C**) The level of *Vp28* expression in the hemocytes (B) and intestine (C) of MK2206-injected shrimp following WSSV infection was analyzed by qPCR at 36 hpi. (**D**) The level of VP28 expression and AKT phosphorylation in the hemocytes of MK2206-injected shrimp challenged with WSSV and analyzed by Western blot. ACTB (β-actin) was used as a loading control; (**d**) Statistical analysis of panel D based on three independent experiments. The subpanels of p-AKT and ACTB were from different gels with same amount of loading samples. (**E**) WSSV copy numbers in the intestines of MK2206-injected shrimp challenged with WSSV analyzed by qPCR at 36 hpi. (**F**) The level of 4EBP1 phosphorylation in the DMSO- or MK2206-injection groups (1250 ng/g body weight) analyzed by Western blot at 36 hpi. The upper panel represents the statistical analysis of three independent experiments of the lower panel. (**G**) The level of S6K1 phosphorylation in the DMSO- or MK2206-injection groups (1250 ng/g body weight) analyzed by Western blot at 36 hpi. The upper panel represents the statistical analysis of three independent experiments of the western blot. The subpanels of p-S6K1 and ACTB were from different gels with same amount of loading samples. (**H**) The survival rate of shrimp following an injection with MK2206 and WSSV compared with the control group. The survival rate of each group was calculated, and the survival curves were presented as Kaplan-Meier plots. Differences between the two groups were analyzed statistically using a log-rank test in GraphPad Prism 8.0. Significant differences were analyzed using a Student’s *t*-test, *P* < 0.05 was considered to indicate a significant difference; different lowercase letters indicate significant differences (*P* < 0.05) in the one-way ANOVA analysis.

To elucidate whether AKT, the upstream molecule of mTORC1, was involved in the activation of mTORC1 signaling in WSSV infection, an injection of MK2206, a specific AKT inhibitor, was administered, and activation of mTORC1 (using phosphorylation of 4EBP1 as the indicator) and WSSV replication (using *Vp28* expression as an indicator) were analyzed. First, the toxicity of MK2206 in shrimp was analyzed, and the results showed that 1250 ng/g MK2206 injection had no significant effect on shrimp mortality (Fig 4A). Next, we injected the shrimp with different doses of MK2206 following WSSV infection and detected the level of *Vp28* expression. The results showed that *Vp28* expression was significantly decreased in the shrimp at three different doses both in the hemocytes and intestines compared with the group injected with DMSO (Fig 4B and 4C). The protein level of VP28 expression and AKT phosphorylation were both decreased in a dose-dependent manner in the MK2206-injection group compared with the DMSO-injection group following WSSV infection (Fig 4D and 4d). The WSSV copy number was also significantly decreased in a concentration-dependent manner in the intestines of shrimp injected with MK2206 (Fig 4E). Next, we detected the levels of 4EBP1 and S6K1 phosphorylation following an injection with MK2206 (1250 ng/g). The results showed that the phosphorylation level of 4EBP1 and S6K1 were significantly decreased in hemocytes compared with the control group (Fig 4F and 4G). Finally, the survival rate of shrimp was also analyzed after injecting shrimp with MK2206 following WSSV infection, and the survival rate of the shrimp increased significantly (Fig 4H), All the results indicated that AKT was indispensable for the activation of mTORC1 signaling in WSSV-infected shrimp.

### WSSV activates the mTORC1 signaling pathway via its receptor, pIgR mediated infection

**Fig 5:**
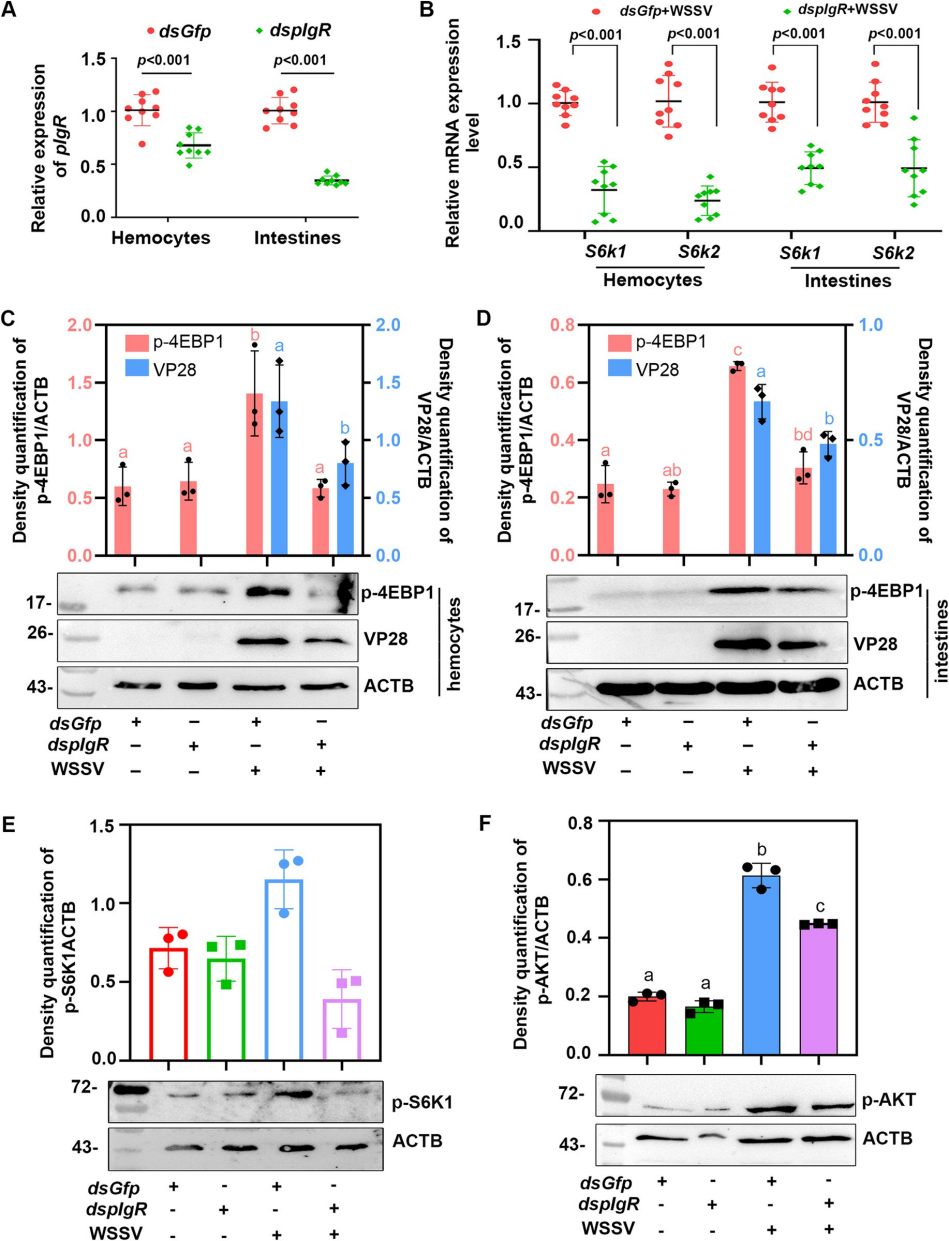
WSSV infection activates the mTORC1 signaling pathway through viral receptor, pIgR. (**A**) The efficiency of *pIgR*-RNAi in the hemocytes and intestines of shrimp analyzed by qPCR at 24 h post dsRNA injection. An injection with *dsGfp* was used as the control. (**B**) The level of mRNA expression of the downstream effectors of mTORC1, *S6k1* and *S6k2*, in the hemocytes of *pIgR*-knockdown shrimp, based on three independent experiments analyzed by qPCR at 36 hpi. (**C** and **D**) The level of VP28 expression and 4EBP1 phosphorylation in the hemocytes (C) and intestines (D) of *pIgR*-RNAi shrimp with or without WSSV infection as analyzed by Western blot at 36 hpi. The upper panel represents the statistical analysis of three independent experiments of the lower panel. (**E**) The level of p-S6K1 in the hemocytes of *pIgR*-RNAi shrimp with or without WSSV challenge analyzed by Western blot at 36 hpi. The upper panel represents the statistical analysis of three independent western blot analyses. (**F**) The level of AKT phosphorylation in the hemocytes of *pIgR*-RNAi shrimp with or without WSSV challenge analyzed by Western blot at 36 hpi. The upper panel represents the statistical analysis of three independent experiments of the lower panel. In panel C-F, the subpanels of VP28/p-S6K1/pAKT and ACTB were from different gels with same amount of loading samples. Significant differences were analyzed using a Student’s *t*-test, *P* < 0.05 was considered to indicate a significant difference; different lowercase letters indicate significant differences (*P* < 0.05) in the one-way ANOVA analysis.

Although several studies have reported that WSSV infection activates the mTOR signaling, it is not clear how WSSV activates this pathway. Thus, it is of interest to identify the viral factors involved in the activation of this pathway. Our previous research found that pIgR is a receptor of WSSV for viral infection via the pIgR-CaM-Catherin endocytosis pathway [27]. To further explore whether WSSV infection activates the mTOR signaling pathway through pIgR, *pIgR*-RNAi was performed and the expression of the mTORC1 target genes, *S6ks*, was analyzed. The results showed that after knocking down *pIgR* expression in the hemocytes and intestines (Fig 5A), the level of *S6k1* and *S6k2* mRNA expression decreased significantly (Fig 5B). We also analyzed phosphorylation of 4EBP1 (another mTORC1 target protein) and found that p-4EBP1 was significantly decreased in *pIgR*-RNAi shrimp following WSSV infection, and there were no obvious changes of p-4EBP1 in the shrimp without WSSV infection (Fig 5C and 5D). At the same time, the protein level of VP28 expression was significantly decreased in the hemocytes and intestines in *pIgR*-RNAi shrimp infected with WSSV (Fig 5C and 5D). And we also detected the phosphorylation levels of S6K1 and AKT in the *pIgR*-RNAi shrimp and found the levels of S6K1 and AKT phosphorylation were significantly decreased in the *pIgR*-RNAi shrimp compared with the *Gfp*-RNAi group following WSSV infection, and there were no obvious changes in S6K1 (Fig 5E) and AKT (Fig 5F) phosphorylation in the *pIgR*-RNAi shrimp in the absence of WSSV infection. Meanwhile, we performed RNAi of *β-Integrin* (another WSSV receptor) [26] and detected the activation of mTORC1 by analyzing phosphorylation of 4EBP1 and WSSV replication in the shrimp. As is shown in S7 Fig, although WSSV replication was inhibited, p-4EBP1 level was not changed obviously in *β-Integrin*-RNAi shrimp. This result suggests β-Integrin mediated WSSV endocytosis did not use mTORC1 signaling for the viral proliferation. These results suggested that pIgR, as a receptor of WSSV, is involved in the activation of the mTORC1 signaling pathway in shrimp infected with WSSV.

### The injection of recombinant VP24 activates mTORC1 signaling

**Fig 6:**
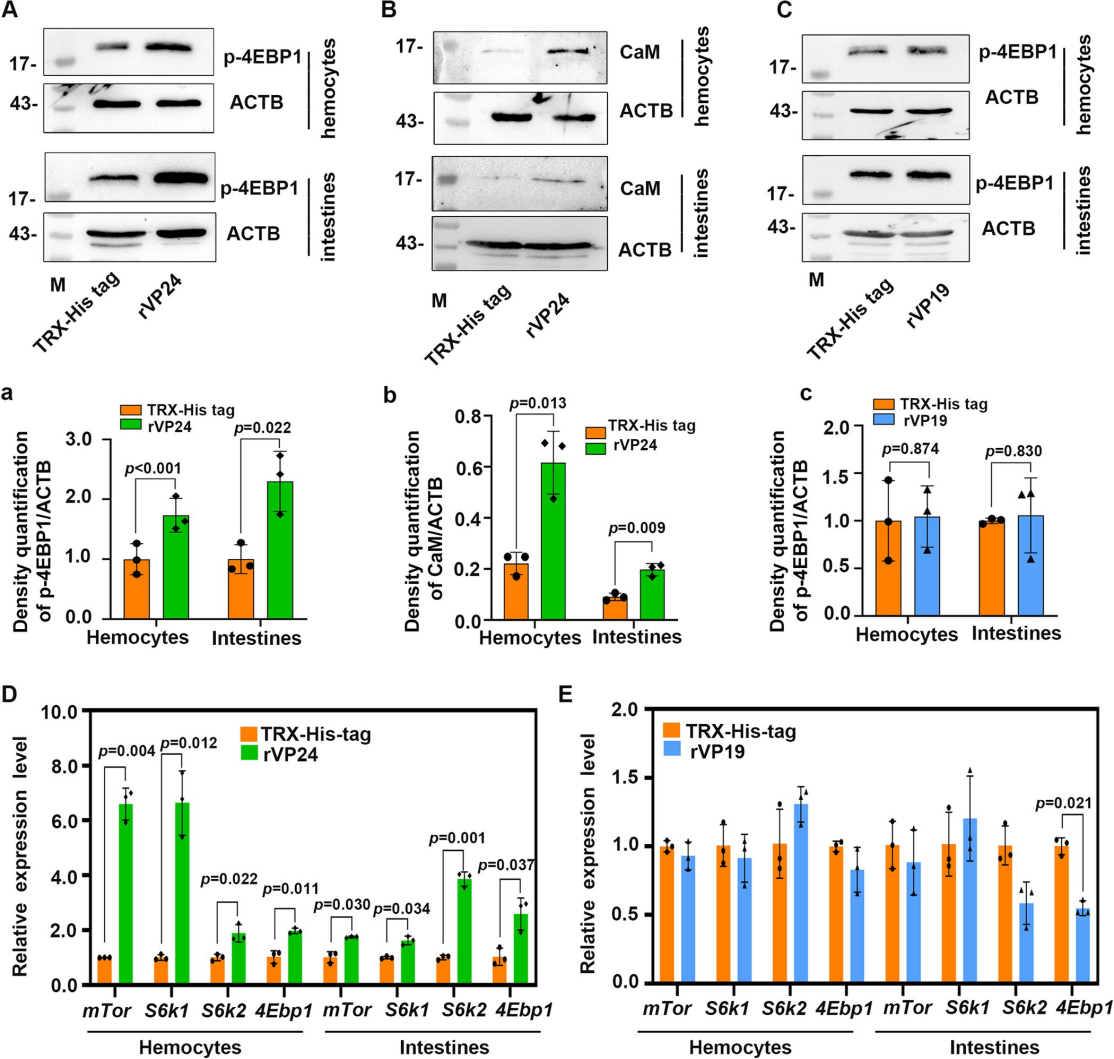
Recombinant VP24 can activate mTORC1 signaling via interacting with pIgR. (**A**) The phosphorylation of 4EBP1 in the hemocytes and intestines of shrimp injected with rVP24 or TRX-His tag analyzed by Western blot at 24 h post protein injection; (**a**) Statistical analysis of panel B based on three independent experiments. (**B**) The level of CaM protein expression in the hemocytes and intestines of shrimp injected with rVP24 or TRX-His tag analyzed by Western blot at 24hpi; (**b**) Statistical analysis of panel B based on three independent experiments. (**C**) The level of 4EBP1 phosphorylation in the hemocytes and intestines of shrimp injected with rVP19 or TRX-His tag analyzed by Western blot at 24 hpi; (**c**) Statistical analysis of panel C based on three independent experiments. (**D** and **E**) The relative expression level of *mTor*, *S6k1*, *S6k2*, and *4Ebp1* mRNA in the hemocytes and intestines of shrimp injected with rVP24 (D) or rVP19 (E) compared with the control group (TRX-His-tag) analyzed by qPCR at 24 hpi. Significant differences were analyzed using a Student’s *t*-test, and *P* < 0.05 was considered to indicate a significant difference.

The receptor, pIgR, interacts with the WSSV envelope protein, VP24, to mediate viral endocytosis [27]. To further confirm WSSV-mediated activation of mTORC1 signaling via the pIgR receptor, an injection of recombinant VP24 was performed, and the mTORC1 activation was analyzed by detecting expression and/or phosphorylation of mTOR, S6K1s, 4EBP1, and calmodulin (CaM). We first expressed VP24 and TRX-His-tag (for control) in *Escherichia coli* (S8A and S8B Fig) and removed endotoxin (LPS) with Triton X-114. Following an injection with the rVP24 or controls (rVP19 and TRX-His tag), the injected proteins in hemocytes were detected by immunocytochemical assays (S9A Fig), and the phosphorylation levels of 4EBP1 and S6K1 were also analyzed. The results showed that the level of 4EBP1 phosphorylation was significantly increased in the hemocytes and intestines of the rVP24 injection group compared with the control group, shrimp injected with TRX-His tag (Fig 6A and 6a), the level of S6K1 phosphorylation was also significantly increased in the hemocytes compared with the control group (S9B Fig). CaM can bind to *pIgR* and promote the entry of WSSV in shrimp [27]. Therefore, we detected the level of CaM protein expression. The results showed that CaM was significantly increased in the rVP24 injection group compared with the control group in the hemocytes and intestines (Fig 6B and 6b). To further corroborate the reliability of these results, we performed a similar experiment using rVP19 expressed in *E*. *coli* (S8C Fig), another WSSV envelope protein which does not bind to pIgR. We found that the level of 4EBP1 phosphorylation in shrimp did not change significantly following an injection with rVP19 (Fig 6C and 6c), and there was also no significant change in phosphorylation of S6K1 (S9C Fig). In addition, the levels of *mTor*, *S6k1*, *S6k2*, and *4Ebp1* mRNA expression were detected after injection of rVP24 or rVP19 into shrimp for 24 h, the results showed that in the rVP24 injection group, the levels of *mTor*, *S6k1*, *S6k2* and *4Ebp1* mRNA expression significantly increased compared with control group (Fig 6D), but no change in rVP19 injection group (Fig 6E). These findings indicated that similar to an WSSV infection, VP24 can activate mTORC1 signaling, however, rVP19 cannot activate mTORC1 signaling. Moreover, VP24 mediates the pIgR-CaM endocytic pathway to activate mTORC1 signaling in shrimp.

### Calmodulin is involved in AKT phosphorylation and activation of mTORC1 signaling

**Fig 7:**
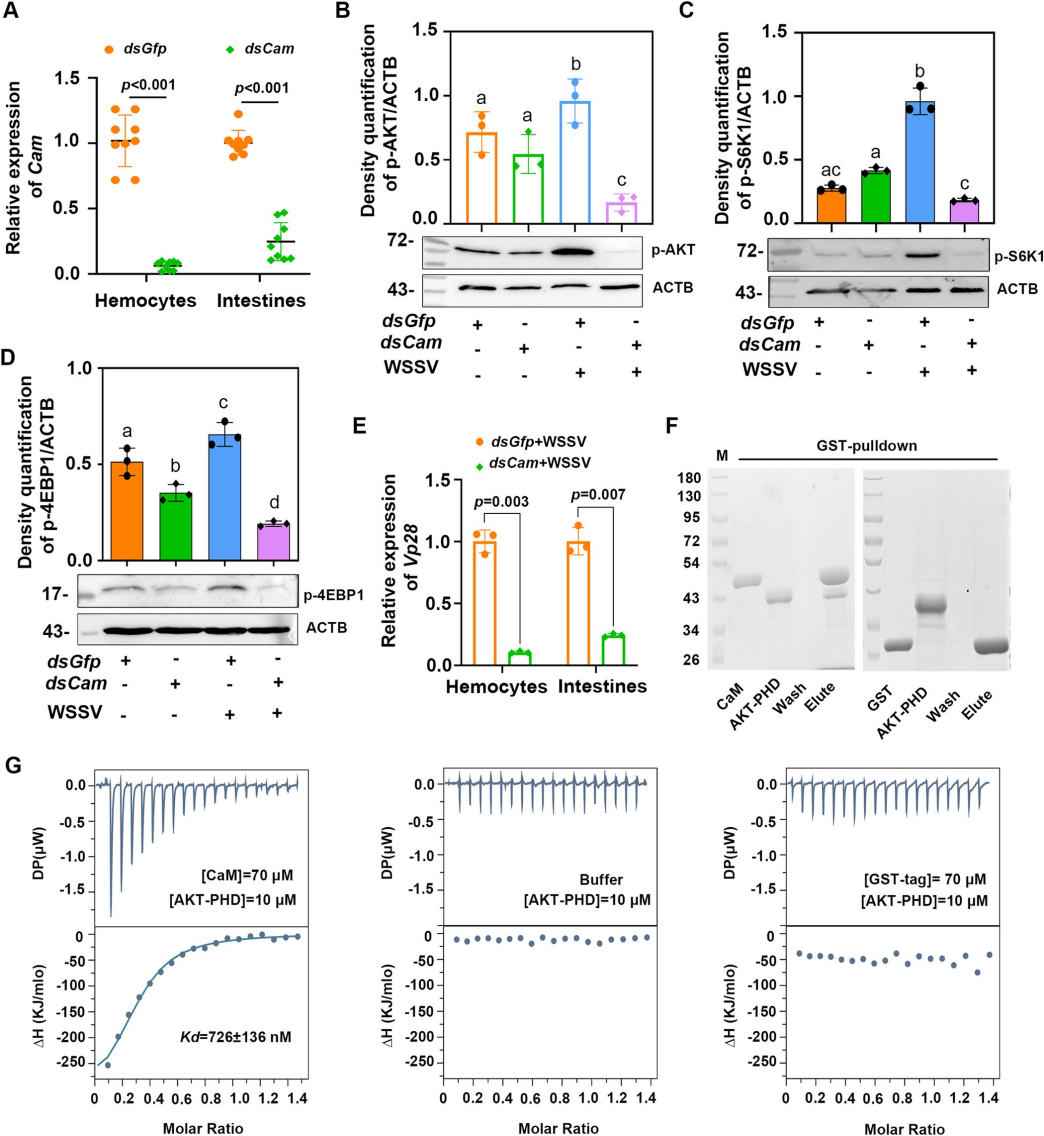
WSSV infection activates mTORC1 signaling via the pIgR-CaM-AKT cascade in shrimp. (**A**) Efficiency of *Cam*-RNAi in the hemocytes and intestines of shrimp analyzed by qPCR at 36 h post dsRNA injection. (**B**) The level of AKT phosphorylation in the hemocytes of *Cam*-RNAi shrimp with or without WSSV infection analyzed by Western blot at 36 hpi. (**C**) Phosphorylation of S6K1 in the hemocytes of *Cam*-RNAi shrimp with or without WSSV infection analyzed by Western blot at 36 hpi. In panel B and C, the subpanels of pAKT/p-S6K1 and ACTB were from different gels with same amount of loading samples. (**D**) Phosphorylation of 4EBP1 in the intestines of *Cam*-RNAi shrimp with or without WSSV infection analyzed by Western blot at 36 hpi. (**E**) The level of *Vp28* mRNA expression in the hemocytes and intestines of *Cam*-RNAi shrimp challenged by WSSV analyzed by qPCR at 36 hpi. (**F**) Interactions between CaM and AKT-PHD were detected using GST pull-down assays using GST tag as a control. (**G**) Measurements of the binding affinity between CaM and AKT-PHD by ITC. The upper panels show the differential heat released following baseline subtraction of CaM and AKT (left), control buffer (middle), GST and AKT (right). The lower panels show the ITC binding curves, indicating the amount of heat released per mole of CaM-AKT (left), buffer (middle), and GST-AKT (right). The calculated binding affinities (*Kd*) based on the ITC binding curves are shown in the figure. Significant differences were analyzed using a Student’s *t*-test, and *P* < 0.05 was considered to indicate a significant difference.

It has been reported that CaM can bind to AKT and facilitate its translocation to menbrane, which is a critical step in its activation [32–34]. To explore the mechanism by which WSSV infection activates mTORC1 signaling, *Cam*-RNAi was performed, AKT and 4EBP1 phosphorylation was analyzed. Following a knockdown of *Cam* in the hemocytes and intestines (Fig 7A), The levels of AKT and S6K1 phosphorylation in the *Cam*-knockdown shrimp was inhibited in the shrimp infected with WSSV; however, there was no significant decline in the shrimp not infected with WSSV (Fig 7B and 7C). The phosphorylation of 4EBP1 was also significantly decreased in WSSV-infected shrimp. A reduction in the level of 4EBP1 phosphorylation was also observed in the WSSV-uninfected shrimp compared with the *dsGfp*-injection shrimp (Fig 7D). At the same time, the level *Vp28* mRNA was significantly decreased in the hemocytes and intestines of the shrimp compared with that of the *dsGfp*-injection group (Fig 7E). Next, we analyzed the interaction between CaM and AKT using a pulldown assay with the recombinant GST-tagged CaM and His-tagged pleckstrin homology (PH) domain of AKT (AKT-PHD) expressed in *E*. *coli* (S8D and S8E Fig). The results showed that CaM interacted with the pleckstrin homology (PH) domain of AKT in vitro, and there was no interaction between the GST tag with AKT (Fig 7F). To further verify this interaction, we conducted isothermal titration calorimetry (ITC) to measure the *Kd* values for the binding of CaM to Akt-PHD. We obtained ITC data upon the titration of CaM into AKT-PHD under the same buffer conditions using a GST tag protein as a control. As shown in Fig 7G, the CaM interacted with Akt-PHD with a dissociation constant of *Kd* = 726 ± 136 nM, ΔH = -335 ± 25.5 KJ/mol, and -ΔG = -35.1 KJ/mol. These results suggested that WSSV infection activates mTORC1 signaling via the pIgR-CaM-AKT cascade in shrimp.

### WSSV infection promotes viral protein translation and impedes host global protein translation in shrimp

Previous studies have shown that mTORC1 is the major regulator of protein translation in eucaryotes. Since viruses do not have their own translation machinery, the host’s translation machinery is required for the translation of viral proteins [35–36]. To investigate the effect of WSSV infection on the global protein translation in shrimp, we labeled newly synthesized proteins with puromycin (S10A Fig), and then detected the global protein translation in shrimp by Western blot using an anti-puromycin monoclonal antibody. Different concentrations of puromycin were used to label the newly synthesized global proteins in shrimp. The content of the puromycin-labeled proteins increased with increasing concentrations of puromycin in shrimp (S10B Fig). Finally, 1 μg/g shrimp was used for protein labeling. The results showed that newly synthesized proteins in intestines of shrimp infected with WSSV were significantly lower than that of shrimp injected with PBS when VP28 was synthesized in a large number (S10C and S10c Fig). Together, these results suggest that an infection with WSSV can suppress the translation of host global proteins in shrimp and enhance the translation of the viral proteins probably via mTORC1 signaling, and this needs further study.

**Fig 8:**
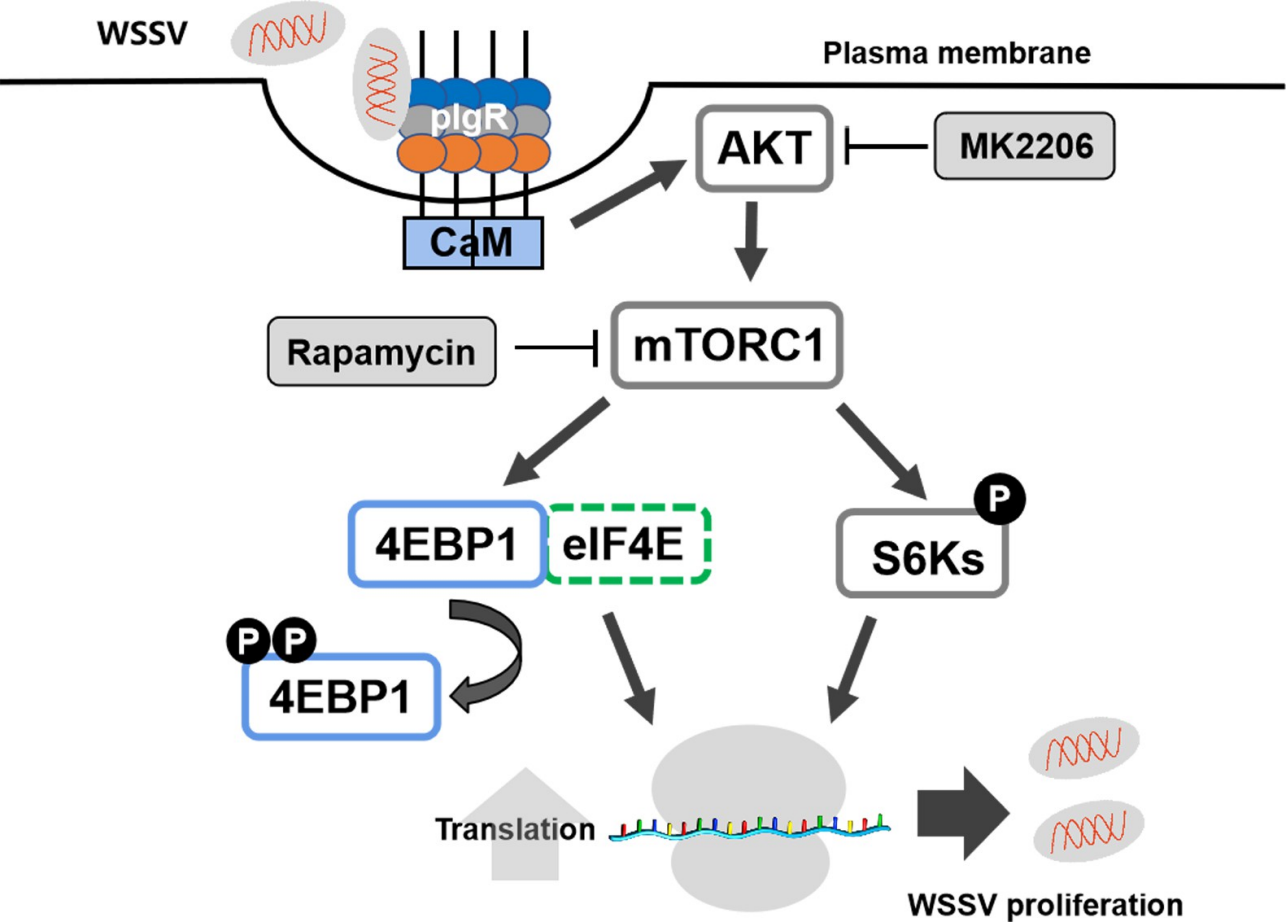
Schematic representation of WSSV-mediated activation of the mTORC1 signaling pathway by the pIgR-CaM-AKT-mTORC1 signalling cascade. WSSV binds to the extracellular domain of pIgR to facilitate the endocytosis of virions into cells. CaM, working as bridge, interacts with the receptor and PH domain of AKT, and promotes AKT activation. Activated AKT then promotes the activation of mTORC1 pathway. mTORC1 further promotes viral DNA and protein synthesis by activating its downstream effectors, 4EBP1, S6K1, and S6K2, and finally promotes WSSV proliferation.

All of the above results suggest that WSSV infection directly activates mTORC1 signaling via the VP24-pIgR-CaM-AKT axis for viral proliferation in shrimp (Fig 8).

## Discussion

In this study, we found that WSSV infection activated the mTORC1 signaling pathway to promote its replication via pIgR-mediated infection in shrimp. The WSSV VP24 protein was found to interact with pIgR and recruited CaM. The latter bound to the PH domain of AKT and induced AKT activation, which led to the activation of mTORC1 signaling and promoted WSSV proliferation via the downstream target proteins, 4EBP1 and S6K1, for viral protein translation in shrimp. Our study further clarified that the viral factor, VP24, of WSSV was involved in the activation of this pathway through the pIgR receptor. In our opinion, this is the first study to reveal how WSSV directly activates mTORC1 signaling via its envelope protein, VP24, in animals.

mTORC1 plays a crucial role in promoting protein synthesis in eucaryotes [9,37]. Moreover, the translation of viral proteins in the host is an important process through which a virus can successfully infect a host. Several different viruses can hijack the mTORC1 signaling pathway and enhance downstream 4EBP1 phosphorylation and S6K1 activity to facilitate their own replication within the host [17]. For example, HPV infection induces increased 4EBP1 phosphorylation [21,38], and HCV infection directly promotes the phosphorylation of 4EBP1 [20]. In *Litopenaeus vannamei*, WSSV infection altered the host metabolome via the PI3K-Akt-mTOR pathway and achieved successful viral replication [29]. On the other hand, mTORC1 signaling can also restrict viral infection [21–22,39]. Previous studies have found that WSSV infection activates the PI3K-Akt-mTOR pathway to promote viral replication in hemocytes at the WSSV genome replication stage (12 hpi) by triggering aerobic glycolysis to provide energy and biosynthetic building blocks in shrimp [29]. WSSV infection can also use the PI3K-Akt-mTOR-HIF1α pathway to induce lipid biosynthesis at 24 h post-viral infection to support WSSV morphogenesis [28]. However, the mechanism by which WSSV activates the AKT-mTOR signaling pathway and which viral factors are involved in the activation of the mTORC1 pathway remain unknown. In our previous study, we found that WSSV utilizes pIgR as a receptor for its infection through an interaction between its envelope protein, VP24. In addition, the pIgR intracellular domain recruits CaM to promote WSSV endocytosis into host cells [27]. Several studies have demonstrated that CaM can directly bind to the PH domain of AKT to induce the AKT translocation to the plasma membrane and ultimately promote the activation of AKT [32–33,40]. Activation of AKT in the plasma membrane is the key to initiating downstream AKT signaling pathways, including mTORC1 signaling [41]. To further explore how WSSV activates mTORC1 signaling, we knocked down the expression of *pIgR*, analyzed phosphorylation of 4EBP1 and S6K1, and found that 4EBP1 and S6K1 phosphorylation were inhibited in the *pIgR*-knockdown shrimp (Fig 5C and 5E). The same results were obtained following the knockdown of *Cam* (Fig 7C and 7D). Moreover, WSSV replication was also inhibited. We also found that CaM directly interacted with the PH domain of AKT (Fig 7F and 7G) and induced activation of AKT. The phosphorylated AKT activated mTORC1. Therefore, all of these results suggest that WSSV can directly activate mTORC1 signaling via the pIgR-CaM-AKT axis, and the WSSV envelope protein, VP24, is involved in activation of mTORC1 signaling.

mTORC1 signaling promotes protein synthesis via the phosphorylation of two kinds of key effectors, S6Ks and 4EBP1. S6Ks are well-characterized targets of mTORC1. mTORC1 directly phosphorylates S6Ks, and subsequently S6Ks phosphorylates and activates several substrates that promote mRNA translation initiation [42]. In mammals, there are two homologs of S6K: S6K1 and S6K2 with high structural similarities and sharing redundant functions [30,43]. For example, the two S6K genes in mice seem to compensate each other, the expression of S6K2 in S6K1 knockout mice will increase, restoring the phosphorylation of rpS6 close to that of wild-type mice in the detected tissues [44]. Recent studies, however, challenge this notion, they may also exhibit distinct functions. For example, S6K2 regulates cancer cell survival via different routes [45]. In the present study, we have identified two homologous proteins of S6K, named S6K1 and S6K2 in *M*. *japonicus* (S6 Fig). We found that whether knockdown of *S6k1* or *S6k2*, the replication of WSSV was both inhibited, and no obvious compensation of the two genes was detected. We also found no off-target effect of *S6k1* RNAi and *S6k2* RNAi (S6C and S6D Fig). These might be that S6K1 and S6K2 regulate protein synthesis by different routes, which needs further study.

The 4EBP1 is the substrate of mTORC1, which is unrelated to S6Ks [14,46]. It inhibits translation by binding eIF4E to prevent assembly of the eIF4F complex. After phosphorylated by mTORC1, 4EBP1 triggers its dissociation from eIF4E [13,47], and promotes translation of 5’cap-dependent mRNAs, which involve in protein synthesis [48]. In our study, we found that WSSV infection increased phosphorylation of 4EBP1 (Fig 2A and 2B), knockdown of *Raptor*, the specific component of mTORC1 following WSSV infection, the phosphorylation of 4EBP1 decreased significantly (S5C Fig), and the proliferation of WSSV was decreased significantly (S5D Fig). These results suggest that as a downstream target of mTORC1, 4EBP1 involves in WSSV replication by promoting viral protein translation.

The results of the present study may indicate that mTORC1 is not the only signaling mechanism involved in WSSV replication. As shown in Fig 2E and 2F, the declined expression of *Ie1* and *Vp2*8, although showed significant difference, a moderate decrease was observed in the shrimp injected with rapamycin, the inhibitor of mTORC1 compared with control. This might suggest that inhibition of mTORC1 alone is not sufficient to reduce WSSV replication completely, and other signaling pathways (e.g., mTORC2) might be involved in WSSV replication.

Viruses can usurp the host translation machinery, targeting almost all steps in the process to that ensure viral protein synthesis is achieved for viral replication [49]. Some viral proteinases can interact with host proteins (i.e., host translation machinery proteins) to inhibit host protein translation [50]. For example, caliciviruses can encode proteases that cleave eIF4G to suppress cap-dependent translation [51]. Other viruses depend on cap-dependent translation and stimulate eIF4F activity by promoting mTOR and eIF4E phosphorylation, as well as the assembly of translation machinery [49]. One recent study found that during Newcastle Disease virus (NDV) infection, viral mRNAs are efficiently translated, whereas host cellular protein synthesis is almost completely inhibited. This effect was attributed to the ability of NDV to activate the PI3K/Akt/mTOR and p38 MAPK/Mnk1 pathways to promote viral mRNA translation via interactions between the viral NP protein and host eIF4E [52]. Similar results were obtained in the present study; there was an observed inhibition of host protein translation and a significant increase in the translation of viral proteins in shrimp following WSSV infection (S10 Fig). The possible mechanisms by which WSSV mediates these effects require further study.

In summary, we identified the VP24-pIgR-CaM-AKT-mTORC1 signaling cascade as a novel pathway by which WSSV facilitates its proliferation in shrimp (Fig 8). WSSV entered host cells via the binding of pIgR through VP24 and inducing the interaction between the pIgR intracellular domain with CaM [27]. Upon interacting with the PH domain of AKT, CaM promoted the phosphorylation of AKT, thereby activating the downstream mTORC1 signaling pathway to promote viral DNA and protein synthesis through 4EBP1, S6K1 and S6K2, the target proteins of mTORC1 in shrimp.

## Materials and methods

### Ethics statement

The rabbit experiments for antibody preparation in the study were carried out in accordance with protocols approved by the Animal Care & Welfare Committee at Shandong University School of Life Sciences (SYDWLL-2021-53).

### Animals

Healthy *M*. *japonicus* (9 g—12 g each) were purchased from a market in Qingdao, Shandong, China. The animals were cultured in a laboratory shrimp culture system with natural sea water for at least 24 h to acclimate the culture system. The animals were randomly selected for the following experiments. Each of the sample was extracted using tissues from 3–5 shrimp and all the experiment were performed three times independently.

### WSSV challenge and tissue collection

The WSSV inoculum was prepared based on previously described methods and quantitative real-time PCR (qPCR) was used for viral quantification [53]. Each shrimp was injected with 50 μL WSSV virions (1 × 10^6^) for infection. The same volume of sterile phosphate-buffered saline (PBS) (140 mM NaCl, 2.7 mM KCl, 10 mM Na_2_HPO_4_, 1.8 mM KH_2_PO_4_, pH 7.4) was injected into the control groups. Hemolymph was extracted from 3 or 4 shrimp using a sterile syringe with anticoagulant buffer (450 mM NaCl, 10 mM KCl, 10 mM EDTA, 100 mM HEPES, pH 7.45) and centrifuged at 800 × g for 6 min at 4°C. Hemocytes were collected for further experiments. Other tissues were dissected with scissors and forceps on ice from at least three shrimp for RNA or protein extraction.

### RNA extraction, cDNA synthesis, DNA and protein extraction

Total RNA was isolated from the hemocytes and different organs (heart, hepatopancreas, gills, stomachs, and intestines) of shrimp using TRIzol (ET101, Transgen, Beijing, China). First strand cDNA synthesis was performed using a cDNA Synthesis Kit (5×All-in-One RT MasterMix; Applied Biological Materials-abm, Vancouver, Canada) in accordance with the manufacturer’s instructions. Genomic DNA was extracted using a Genomic DNA Purufication Kit (Toyobo, Osaka, Japan). Protein samples from different organs and hemocytes were obtained by separately homogenizing in radio-immunoprecipitation assay (RIPA) buffer (150 mM NaCl, 0.1% SDS, 1% Triton X-100, 1% EDTA, 50 mM Tris-HCl, pH 7.8). The homogenates were centrifuged at 12000 × g for 10 min at 4°C and the supernatant was collected for further analysis.

### Tissue distribution and *mTor* expression profiles

The total cDNA sequences for *mTor* from the shrimp were obtained from the transcriptome sequencing of *M*. *japonicus* [54]. The tissue distribution of *mTor* mRNA was determined by semi-quantitative reverse transcription-PCR (RT-PCR) using *mTor*-RT-F and *mTor*-RT-R primers (S1 Table). *β-actin* was used as the internal control, with *β-actin*-RT-F and *β-actin*-RT-R primers. The PCR procedure consisted of an initial incubation at 94°C for 3 min; followed by 32 cycles of 94°C for 30 s, 52°C for 30 s, and 72°C for 30 s; followed by 72°C for 10 min. The PCR products were analyzed using agarose gel electrophoresis (1.5% agarose).

Quantitative real-time PCR (qPCR) was performed to determine the expression profiles of *mTor* in the shrimp following a challenge with WSSV using the above primers. qPCR was performed as follows: 95°C for 10 min; 40 cycles at 95°C for 10 s, and 60°C for 50 s; followed by a melting period from 65°C to 95°C. The obtained data were analyzed using the cycle threshold (2^−ΔΔCT^) method [55]. *β-actin* and the *elongation factor-1α* (*Ef-1α*) were used as the internal controls with primers β-actin-RT-F, β-actin-RT-R and *Ef-1α-*F, and *Ef-1α-*R, respectively. The results were expressed as the mean ± SD from three independent replicates.

### Bioinformatic analysis

The total cDNA sequences for *mTor*, *Akt*, *S6k1*, *S6k2*, and *4Ebp1* were all obtained from the transcriptome sequencing of *M*. *japonicus* and the sequences were confirmed by RT-PCR. The amino acid sequences, theoretical molecular weights, and isoelectric points of the above molecules were analyzed using an online server (http://web.expasy.org/translate/). A domain prediction tool (SMART: http://smart.emblheidelberg.de/) was used to analyze the protein domain architecture. Phylogenetic trees of the molecules from different species were constructed using MEGA7 (Download from https://www.megasoftware.net/).

### Western blotting

The proteins extracted from different organs or hemocytes were separated using 10% sodium dodecyl sulfate polyacrylamide gel electrophoresis (SDS-PAGE) and transferred onto a nitrocellulose membrane via electrical transfer in transfer buffer (25 mM Tris, 193 mM Glycine, 0.037% SDS, 20% C_2_H_5_OH). After blocking with 5% nonfat milk or 3% bovine serum albumin (BSA) diluted in TBS buffer (150 mM NaCl, 3 mM EDTA, 50 mM Tris-HCl, pH 8.0) for 1 h, the membranes were incubated with the following primary antibodies: anti-*β*actin (ACTB) at a 1:250 dilution, anti-VP28 at a 1:200 dilution, anti-CaM at 1:100 dilution (prepared in our laboratory); anti-phosphorylated (p)-4EBP1 (Thr37/46) monoclonal antibodies (Catalog No. 2855, Cell Signaling Technology, America, 1:1000); anti-phosphorylated (p)-AKT (Ser473) polyclonal antibodies (Catalog No. #11054, Signalway Antibody, USA, 1:500); anti-phosphorylates (p)-S6K1 (Thr389) (Catalog No. AP0564, ABclonal, Wuhan, China, 1:500); anti-puromycin (Catalog No. EQ0001, Kerafast, 1:1000), and gently shaken overnight at 4°C. After washing three times with TBST (0.1% Tween-20 added to TBS), the membranes were incubated with alkaline phosphatase goat anti-rabbit antibodies (ZB2308 ZSGB-Bio, Beijing, China, 1:5,000), horseradish peroxidase-conjugated goat anti-rabbit antibodies (ZB2301 ZSGB-Bio, Beijing, China, 1:5,000), or peroxidase-conjugated goat anti-mouse antibodies (ZB2305 ZSGB-Bio, Beijing, China, 1:5,000) for 3 h at room temperature with gentle shaking. The immunoreactive protein bands were developed using a Nitrotetrazolium blue chloride (A610379, BBI) and P-toluidine salt (A610072, BBI) solution in the dark or using enhanced chemiluminescence (ECL). The protein bands were digitalized using Image J software and statistically analyzed with GraphPad Prism 8.0.

### RNA interference (RNAi)

RNAi was performed to analyze the function of mTOR. Gene-specific primers *dsmTor*-F and *dsmTor*-R were linked to the T7 promoter (S1 Table) and used to amplify a partial sequence of *mTor* cDNA. The PCR products were used as the templates for double-stranded RNA (dsRNA) synthesis using T7 RNA polymerase (Fermentas, Burlington, Canada), following the manufacturer’s instructions. The *dsGfp* (green fluorescent protein) coding region, serving as a control, was amplified using the *dsGfp*-F and *dsGfp*-R primers (S1 Table) and synthesized using the above method. For the RNAi assay, shrimp were randomly divided two groups (20 shrimp/group), and *dsmTor* (5 μg/g) was injected into the muscles of shrimp using a 50 μL syringe. The same dose of dsRNA was injected again at 12 h following the first injection. The same dose of *dsGfp* was used as a control. RNAi efficiency was assessed after 24 h using qPCR. Similar methods were used to knockdown *Raptor*, *Rictor*, *S6k1*, *S6k2*, *pIgR*, *Cam* and *β-Integrin*.

### Rapamycin injection

The mTOR inhibitor, rapamycin (Aladdin, Shanghai, China) was dissolved in DMSO to create a highly concentrated storage solution (10 mg/mL). When using, the storage solution was first diluted to 40 μg/mL with DMSO, and finally, 4 μg/mL of the required injection solution was diluted with PBS. The shrimp were divided into two groups (15 shrimp/group). The rapamycin solution (50 μL) was injected into each shrimp at the penultimate segment. The final concentration of the rapamycin solution injected into the shrimp was approximately 20 ng/g shrimp. The control group was injected with same volume of PBS containing 10% DMSO.

### Survival rate assay

The survival rate was analyzed following *mTor*-RNAi in shrimp challenged with WSSV. The shrimp were divided into two groups (40 shrimp/group): 1) *dsGfp* group; and 2) *dsmTor* group. After administering RNAi for 24 h, an inoculum of WSSV (1 × 10^5^ copies) was separately injected into two groups of shrimp. The number of dead shrimp in the two groups were observed every 12 h after infection, the survival rate of each group was calculated, and the survival curves were presented as Kaplan-Meier plots. Differences between the two groups were analyzed with a log-rank test using GraphPad Prism 8.0 software. Significant differences were considered at a threshold of *P* < 0.05.

The survival rate of the rapamycin-injected shrimp was also analyzed. The shrimp were randomly divided into two groups (35 individuals/group): 1) the rapamycin-injected group; and 2) the DMSO-injected group. At 2 h following rapamycin injection, WSSV (1 × 10^5^) was separately injected into two groups of shrimp. The number of dead shrimp in the two groups was observed every 12 h after infection, and the dead shrimp were immediately removed. The survival rate was obtained using the above method.

### Phosphorylation analysis of 4EBP1 and S6K1 in shrimp following WSSV infection

To explore whether mTORC1 is activated by WSSV, phosphorylation of 4EBP1 and S6K1 were analyzed by Western blot as an indicator. The shrimp were randomly divided into two groups (20 shrimp each group): one group was injected with WSSV (1 × 10^6^), and a control group was injected with same volume of PBS. Hemocytes and intestines were dissected from the shrimp in each of the two groups at different time points for protein extraction. Western blotting was used to analyze the phosphorylation of 4EBP1 and S6K1 in shrimp using a p-4EBP1 antibody and p-S6K1 antibody.

### Detection of global protein translation in shrimp

As a peptidyl transfer inhibitor, puromycin has been widely used in cell biology to tag newly synthesized proteins [56]. We used puromycin (MACKLIN, Shanghai, China) to analyze the nascent proteins in shrimp challenged by WSSV and PBS. Shrimp were divided into two groups (10 individuals /group), and WSSV (1 × 10^6^) or PBS were injected into shrimp. Proteins were extracted from the shrimp at 12, 24, and 48 h post-injection with WSSV and PBS. One hour before protein extraction, the shrimp in the experimental and control groups were injected with the same dose of puromycin (1 μg/g). A Western blot analysis was performed and puromycin integration into the polypeptide was detected using a puromycin monoclonal antibody. The degree of puromycin integration into the polypeptide reflected the level of global protein translation in the shrimp following the injection with WSSV and PBS.

### Expression and purification of recombinant proteins

The recombinant proteins were induced with 0.5 mM isopropyl-β-D-thiogalactopyranoside at 37°C for 4 h for expression. The GST-tag proteins were purified using affinity chromatography with GST-resin (GenScript, Nanjing, China) in accordance with the manufacturer’s instructions. Recombinant His-tagged proteins were purified by high-affinity Ni-IDA Resin (GenScript). In addition, for the proteins used in vivo (e.g., His-TRX-tag, rVP24, and rVP19), an additional wash in cold 0.1% Triton X-114 was performed to remove any endotoxin contamination [57].

### Pull-down assay

A pull-down assay was performed to explore the interaction between calmodulin (CaM) and AKT protein kinase B. CaM was recombinantly expressed in *E*. *coli* using the recombinant vector, pGEX-4T-1/CaM, and the PH domain of AKT was recombinantly expressed in *E*. *coli* with recombinant vector pET-32a/AKT-PHD. Purified GST-tagged CaM (200 μg) was incubated with the His-tagged AKT-PHD (1:1) overnight at 4°C. Following an incubation with GST-bound resin (100 μL) for 50 min at 4°C, the resin was washed five times with PBS. Elution buffer (10 mM reduced glutathione, 50 mM Tris-HCl, pH 8.0) was added to elute the bound proteins. SDS-PAGE was conducted for protein analysis. A GST-tagged protein was used as a control.

### Isothermal Titration Calorimetry (ITC)

The thermodynamic parameters for CaM binding to AKT-PHD were determined using an Auto-iTC200 microcalorimeter (Malvern Panalytical, Malvern, UK). ITC experiments were performed at 25°C, and the protein samples were suspended in 0.1M Tris-HCl (pH 8.0) buffer. CaM (70 μM) was titrated into a sample cell containing 10 μM AKT-PHD. The heat of the reaction was measured at 25°C for the 19 injections by titrating CaM into the buffer. Data analysis was performed using the Malvern MicroCal PEAQ–ITC Analysis. Baseline corrections were performed by subtracting heat of dilution for the binding of CaM to AKT-PHD. The binding curves were analyzed, and dissociation constants (*Kd*) were determined by nonlinear least square fitting of the baseline-corrected data.

### MK-2206 2HCl injection

The AKT inhibitor, MK-2206 2HCl (AbMole, America), was dissolved in DMSO to make a highly concentrated storage solution (5 mg/mL). The storage solution was first diluted to 2.5 mg/mL with DMSO and finally, the 0.25 mg/mL required injection solution was diluted with PBS. The shrimp were divided into two groups (10 shrimp/group) and the MK-2206 solution (50 μL) was injected into the body at the penultimate segment of the shrimp, after 2 h of inhibitor injection, WSSV (1 × 10^6^) was injected into two groups of shrimp. The final concentration of the MK-2206 solution that was injected into the shrimp was approximately 1250 ng/g shrimp. The control group was injected with PBS containing 10% DMSO.

The survival rate of the MK-2206-injected shrimp was also analyzed. The shrimp were divided into two groups (35 shrimp/group): 1) the MK-2206-injected group; and 2) the DMSO-injected group. At 2 h following the injection with the inhibitor, WSSV (1 × 10^5^) was separately injected into two groups of shrimp. The number of dead shrimp in the two groups was observed every 12 h post-infection, and the dead shrimp were immediately cleaned up. The survival rate was obtained using the methods as described above.

### Statistical analysis

Data were presented as the mean ± standard deviation (SD) of at least three replicates for statistical analysis. Significant differences were analyzed using two-tailed Student’s *t*-test for paired comparisons or a one-way ANOVA for multiple comparisons. *P* value < 0.05 was considered statistically significant. Different lowercase letters indicate significant differences (*P* < 0.05) in the one-way ANOVA analysis. The survival rate was calculated, and the survival curves are presented as Kaplan-Meier plots and the statistically using a log-rank test. All statistical analyses were produced using GraphPad 8.0 data view software. Densitometry analyses of Western blot bands were based on three independent replicates using ImageJ software (National Institutes of Health, http//imagej.nih.gov/ij/download.html).

## Supporting information

S1 Fig Domain architecture of mTORs from different species. *H*. *sapiens*, *Homo sapiens*; *M*. *musculus*, *Mus musculus*; *D*. *rerio*, *Danio rerio*; *D*. *melanogaster*, *Drosophila melanogaster*; *P*. *vannamei*, *Penaeus vannamei*. DUF3385, the uncharacterized domain ranged from 160 to 172 amino acids in length and was identified in the phosphatidylinositol kinase-related protein kinases of mTOR: representatives of the three main groups sharing the domain **F**RAP, **A**TM, and **T**RRAP (FAT); rapamycin binding domain (FRB); kinase, PI3kc kinase; and **F**RAP, **A**TM, **T**RRAP **C**-terminal (FATC).
(TIF)

S2 Fig Alignment of mTOR amino acid sequences from different species. The mTOR sequences were derived from GenBank. *Diachasma alloeum*, XP_015118165.1; *Drosophila melanogaster*, NP_524891.1; *Homo sapiens*, NP_004949.1; *Bombyx mori*, NP_001171773.1; *Danio rerio*, ABG56082.2; *Mus musculus*, NP_064393.2; *Penaeus vannamei*, XP_027228160.1. The domains in the red box represents the kinase and FATC domains, respectively.
(TIF)

S3 Fig Phylogenetic tree of mTORS from different species. The mTOR sequences of different species were obtained from GenBank, and the NJ tree was established using MEGA 6.0. The results were repeated 1000 times by bootstrapping. The mTOR of *M*. *japonicus* is denoted by a black triangle.
(TIF)

S4 Fig *mTor* expression upregulated in shrimp after WSSV challenge. **A.** Tissue distribution of *mTor* in shrimp at the mRNA level detected using RT-PCR. **B-D.** Expression patterns of *mTor* in hemocytes (B), gills (C), and intestines (D), detected by qPCR. β-Actin was used as an internal control. Significant differences were analyzed using a Student’s *t*-test and *P* < 0.05 was accepted as a significant difference.
(TIF)

S5 Fig Phosphorylation of 4EBP1 and AKT regulated by mTORC1 and mTORC2, respectively. **A.** The efficiency of *Raptor* RNAi in hemocytes and intestines in shrimp as detected by qPCR. **B.** The efficiency of *Rictor* RNAi in the hemocytes and intestines detected by qPCR. **C.** 4EBP1 phosphorylation after the knockdown of *Raptor* and *Rictor* in the hemocytes and intestines. **c.** Statistical analysis of three independent experiments for panels C. **D.** The level of VP28 protein expression in the hemocytes and intestines of *Raptor*-RNAi shrimp challenged with WSSV and detected by Western blot at 36 hpi. ACTB was used as the loading control. **d.** Statistical analysis of three independent experiments for panel D.
(TIF)

S6 Fig **A.** Phylogenetic tree of S6K1s and S6K2s from different species. S6K sequences of different species were obtained from GenBank, and an NJ tree was established using MEGA 6.0. **B.** Domains architecture of S6K1 and S6K2 in *M*. *japonicus*. **C.** The mRNA expression level of *S6k2* detected by qPCR after knocking down of *S6k1*. **D.** The mRNA expression level of *S6k1* detected by qPCR after knocking down of *S6k2*.
(TIF)

S7 Fig After knockdown of *β*-Integrin, WSSV replication was inhibited and phosphorylation of 4EBP1 was not changed. **A**. Efficiency of *β-Integrin*-RNAi in the hemocytes and intestines of shrimp analyzed by qPCR. **B-C**. *β-Integrin* knockdown, the expression of *Vp28* (B) and *Ie1* (C) at the transcriptional level detacted by qPCR. **D**. The WSSV copy number decreased significantly after *β-Integrin* knockdown. **E**. Phosphorylation of 4EBP1 was detected after knocking down of *β-Integrin*. **e**. Statistical analysis based on three independent experiments of (E). Significant differences were analyzed using a Student’s *t*-test, and *P* < 0.05 was considered to indicate a significant difference.
(TIF)

S8 Fig Purification of expressed recombinant WSSV envelope proteins, His-TRX Tag, CaM, and AKT-PHD. **A-C.** TRX-His tag **(A)**, rVP24 **(B)**, and rVP19 **(C)** expression and purification from *E*. *coli*. Lane 1, the total proteins from *E*. *coli* with pET32a (+) parental plasmid or pET32a-Vp24 or pET32a-Vp19 without IPTG induction; lane 2, total proteins from *E*. *coli* with IPTG induction; lane 3, purified recombinant proteins (TritonX-114 was used to remove endotoxins for all three of the proteins used in the in vivo injection). **D-E.** CaM **(D)** and AKT-PHD **(E)** expression and purification from *E*. *coli*. Lane 1, total proteins from *E*. *coli* with pGEX4T-1-CaM or pET-32a-AKT-PHD without IPTG induction; lane 2, total proteins from the *E*. *coli* with IPTG induction; lane 3, purified recombinant proteins.
(TIF)

S9 Fig Phosphorylation of S6K1 in hemocytes was detected by injection of recombinant VP24. **A.** Immunocytochemistry was performed to detect the entry of recombinant proteins into hemocytes. DIC, differential interference construct. Scale bar = 20 μm. **B-C.** The phosphorylation of S6K1 in the hemocytes of shrimp injected with rVP24 (B) or rVP19 (C) compared with control group analyzed by Western blot at 24 h post proteins injection. The upper panel represents the statistical analysis of three independent experiments of the lower panel.
(TIF)

S10 Fig Global protein translation is impaired and VP28 translation is enhanced in shrimp following WSSV infection at different time points. **A**. Structural formula of puromycin. **B**. The level of global protein translation labelled with different concentrations of puromycin was detected in shrimp challenged with WSSV. **C**. The global protein and VP28 translation were detected in the intestines of shrimp at different time points post-WSSV and PBS injection by Western blot using anti-puromycin as the primary antibody; **c**. Statistical analysis of panel C based on three independent experiments. Significant differences were analyzed using a Student’s *t*-test, and *P* < 0.05 was considered to indicate a significant difference.
(TIF)

S1 Table Primer sequences used in this article. Annealing temperature (°C). Amplicon size (bp). The amplification efficiency of the primers (%)
(DOCX)

## References

[1] Sabers CJ, Martin MM, Brunn GJ, Williams JM, Dumont FJ, Wiederrecht G, et al. Isolation of a protein target of the FKBP12-rapamycin complex in mammalian cells. Journal of Biological Chemistry. 1995; 270(2): 815–822. doi: 10.1074/jbc.270.2.815 7822316

[2] Helliwell SB, Wagner P, Kunz J, Deuter-Reinhard M, Henriquez R, Hall MN. TOR1 and TOR2 are structurally and functionally similar but not identical phosphatidylinositol kinase homologues in yeast. Molecular biology of the cell. 1994; 5(1): 105–118. doi: 10.1091/mbc.5.1.105 8186460 PMC301013

[3] Xu X, Ye L, Araki K, Ahmed R. mTOR, linking metabolism and immunity. Seminars in Immunology. 2012; 24(6): 429–435. doi: 10.1016/j.smim.2012.12.005 23352227 PMC3582734

[4] Shimobayashi M, Hall MN. Making new contacts: the mTOR network in metabolism and signalling crosstalk. Nature Reviews Molecular Cell Biology. 2014; 15(3): 155–162. doi: 10.1038/nrm3757 24556838

[5] Raught B, Gingras A, Sonenberg N. The Target of Rapamycin (TOR) Proteins. Proc. Natl. Acad. Sci. U. S. A. 2001; 98(13): 7037–7044. doi: 10.1073/pnas.121145898 11416184 PMC34619

[6] Weichhart T, Hengstschläger M, Linke M. Regulation of innate immune cell function by mTOR. Nature Reviews Immunology. 2015; 15(10): 599–614. doi: 10.1038/nri3901 26403194 PMC6095456

[7] Sturgill TW, Cohen A, Diefenbacher M, Trautwein M, Martin DE, Hall MN. TOR1 and TOR2 have distinct locations in live cells. Eukaryotic Cell. 2008; 7(10): 1819–1830. doi: 10.1128/EC.00088-08 18723607 PMC2568074

[8] Sabatini DM. mTOR and cancer: insights into a complex relationship. Nature Reviews Cancer. 2006; 6(9): 729–734. doi: 10.1038/nrc1974 16915295

[9] Ben-Sahra I, Manning BD. mTORC1 signaling and the metabolic control of cell growth. Current Opinion in Cell Biology. 2017; 4572–82. doi: 10.1016/j.ceb.2017.02.012 28411448 PMC5545101

[10] Kim YC, Guan K. mTOR: a pharmacologic target for autophagy regulation. Journal of Clinical Investigation. 2015; 125(1): 25–32. doi: 10.1172/JCI73939 25654547 PMC4382265

[11] Sarbassov DD, Ali SM, Sabatini DM. Growing roles for the mTOR pathway. Current Opinion in Cell Biology. 2005; 17(6): 596–603. doi: 10.1016/j.ceb.2005.09.009 16226444

[12] Magnuson B, Ekim B, Fingar DC. Regulation and function of ribosomal protein S6 kinase (S6K) within mTOR signalling networks. Biochemical Journal. 2012; 441(1): 1–21. doi: 10.1042/BJ20110892 22168436

[13] Mamane Y, Emmanuel Petroulakis LR, Yoshida K, Ler LW, Sonenberg N. eIF4E - from translation to transformation. Oncogene. 2004; 23(18): 3172–3179. doi: 10.1038/sj.onc.1207549 15094766

[14] Burnett PE, Barrow RK, Cohen NA, Snyder SH, Sabatini DM. RAFT1 phosphorylation of the translational regulators p70 S6 kinase and 4E-BP1. Proc. Natl. Acad. Sci. U. S. A. 1998; 95(4): 1432–1437. doi: 10.1073/pnas.95.4.1432 9465032 PMC19032

[15] Sarbassov DD, Ali SM, Kim D, Guertin DA, Latek RR, Erdjument-Bromage H, et al. Rictor, a novel binding partner of mTOR, defines a rapamycin-insensitive and raptor-independent pathway that regulates the cytoskeleton. Current Biology. 2004; 14(14): 1296–1302. doi: 10.1016/j.cub.2004.06.054 15268862

[16] Jacinto E, Loewith R, Schmidt A, Lin S, Rüegg MA, Hall A, et al. Mammalian TOR complex 2 controls the actin cytoskeleton and is rapamycin insensitive. Nature Cell Biology. 2004; 6(11): 1122–1128. doi: 10.1038/ncb1183 15467718

[17] Le Sage V, Cinti A, Amorim R, Mouland A. Adapting the stress response: viral subversion of the mTOR signaling pathway. Viruses. 2016; 8(6): 152–170. doi: 10.3390/v8060152 27231932 PMC4926172

[18] Moody C, Scott R, Amirghahari N, Nathan C, Young L, Dawson C, et al. Modulation of the cell growth regulator mTOR by Epstein-Barr Virus-Encoded LMP2A. Journal of Virology. 2005; 79(9): 5499–5506. doi: 10.1128/JVI.79.9.5499-5506.2005 15827164 PMC1082717

[19] Zhang L, Wu J, Ling MT, Zhao L, Zhao K. The role of the PI3K/Akt/mTOR signalling pathway in human cancers induced by infection with human papillomaviruses. Molecular Cancer. 2015; 14(1): 87–99. doi: 10.1186/s12943-015-0361-x 26022660 PMC4498560

[20] George A, Panda S, Kudmulwar D, Chhatbar SP, Nayak SC, Krishnan HH. Hepatitis C virus NS5A binds to the mRNA cap-binding eukaryotic translation initiation 4F (eIF4F) complex and up-regulates host translation initiation machinery through eIF4E-binding protein 1 inactivation. Journal of Biological Chemistry. 2012; 287(7): 5042–5058. doi: 10.1074/jbc.M111.308916 22184107 PMC3281608

[21] Hopkins KC, Tartell MA, Herrmann C, Hackett BA, Taschuk F, Panda D, et al. Virus-induced translational arrest through 4EBP1/2-dependent decay of 5′-TOP mRNAs restricts viral infection. Proc. Natl. Acad. Sci. U. S. A. 2015; 112(22): E2920–E2929. doi: 10.1073/pnas.1418805112 26038567 PMC4460451

[22] Fekete T, Ágics B, Bencze D, Bene K, Szántó A, Tarr T, et al. Regulation of RLR-mediated antiviral responses of human dendritic cells by mTOR. Frontiers in Immunology. 2020; 11572960–572979. doi: 10.3389/fimmu.2020.572960 33013932 PMC7516067

[23] Li C, Weng S, He J. WSSV-host interaction: Host response and immune evasion. Fish & Shellfish Immunology. 2019; 84558–571. doi: 10.1016/j.fsi.2018.10.043 30352263

[24] Verbruggen B, Bickley LK, van Aerle R, Bateman KS, Stentiford GD, Santos EM, et al. Molecular Mechanisms of White Spot Syndrome Virus Infection and Perspectives on Treatments. Viruses. 2016; 8(1): 23–51. doi: 10.3390/v8010023 26797629 PMC4728583

[25] Wang X, Xu Y, Xu J, Zhao X, Wang J. Collaboration between a Soluble C-Type Lectin and Calreticulin Facilitates White Spot Syndrome Virus Infection in Shrimp. Journal of Immunology. 2014; 193(5): 2106–2117. doi: 10.4049/jimmunol.1400552 25070855

[26] Li DF, Zhang MC, Yang HJ, Zhu YB, Xu X. Beta-integrin mediates WSSV infection. Virology. 2007; 368(1): 122–132. doi: 10.1016/j.virol.2007.06.027 17655902

[27] Niu G, Wang S, Xu J, Yang M, Sun J, He Z, et al. The polymeric immunoglobulin receptor-like protein from *Marsupenaeus japonicus* is a receptor for white spot syndrome virus infection. PLoS Pathogens. 2019; 15(2): e1007558. doi: 10.1371/journal.ppat.1007558 30726286 PMC6380602

[28] Hsieh Y, Chen Y, Li C, Chang Y, Liang S, Lin S, et al. To complete its replication cycle, a shrimp virus changes the population of long chain fatty acids during infection via the PI3K-Akt-mTOR-HIF1α pathway. Developmental and Comparative Immunology. 2015; 53(1): 85–95. doi: 10.1016/j.dci.2015.06.001 26112000

[29] Su MA, Huang YT, Chen IT, Lee DY, Hsieh YC, Li CY, et al. An invertebrate Warburg effect: a shrimp virus achieves successful replication by altering the host metabolome via the PI3K-Akt-mTOR pathway. PLoS Pathogens. 2014; 10(6): e1004196. doi: 10.1371/journal.ppat.1004196 24945378 PMC4055789

[30] Lee-Fruman KK, Kuo CJ, Lippincott J, Terada N, Blenis J. Characterization of S6K2, a novel kinase homologous to S6K1. Oncogene. 1999; 18(36): 5108–5114. doi: 10.1038/sj.onc.1202894 10490847

[31] Saitoh M, Dijke PT, Miyazono K, Ichijo H. Cloning and Characterization of p70S6K Defines a Novel Family of p70 S6 Kinases1. Biochemical and Biophysical Research Communications. 1998; (253): 470–476. doi: 10.1006/bbrc.1998.9784 9878560

[32] Agamasu C, Ghanam RH, Xu F, Sun Y, Chen Y, Saad JS. The interplay between Calmodulin and membrane interactions with the pleckstrin homology domain of Akt. Journal of Biological Chemistry. 2017; 292(1): 251–263. doi: 10.1074/jbc.M116.752816 27872186 PMC5217684

[33] Agamasu C, Ghanam RH, Saad JS. Structural and biophysical characterization of the interactions between Calmodulin and the pleckstrin homology domain of Akt. Journal of Biological Chemistry. 2015; 290(45): 27403–27413. doi: 10.1074/jbc.M115.673939 26391397 PMC4646410

[34] Weako J, Jang H, Keskin O, Nussinov R, Gursoy A. The structural basis of Akt PH domain interaction with calmodulin. Biophysical Journal. 2021; 120(10): 1994–2008. doi: 10.1016/j.bpj.2021.03.018 33775637 PMC8204387

[35] Roberts LO, Jopling CL, Jackson RJ, Willis AE. Viral Strategies to Subvert the Mammalian Translation Machinery. 2009; 90313–367. doi: 10.1016/S1877-1173(09)90009-6 20374746 PMC7102724

[36] Montero H, García-Román R, Mora S. eIF4E as a Control Target for Viruses. Viruses. 2015; 7(2): 739–750. doi: 10.3390/v7020739 25690796 PMC4353914

[37] Roux PP, Topisirovic I. Signaling Pathways Involved in the Regulation of mRNA Translation. Molecular and Cellular Biology. 2018; 38(12): e18–e70. doi: 10.1128/MCB.00070-18 29610153 PMC5974435

[38] Asimomytis A, Karanikou M, Rodolakis A, Vaiopoulou A, Tsetsa P, Creatsas G, et al. mTOR downstream effectors, 4EBP1 and eIF4E, are overexpressed and associated with HPV status in precancerous lesions and carcinomas of the uterine cervix. Oncology Letters. 2016; 12(5): 3234–3240. doi: 10.3892/ol.2016.5056 27899988 PMC5103924

[39] McNulty S, Flint M, Nichol ST, Spiropoulou CF. Host mTORC1 Signaling Regulates Andes Virus Replication. Journal of Virology. 2012; 87(2): 912–922. doi: 10.1128/JVI.02415-12 23135723 PMC3554081

[40] Dong B, Valencia CA, Liu R. Ca^2+^/Calmodulin Directly Interacts with the Pleckstrin Homology Domain of AKT1. Journal of Biological Chemistry. 2007; 282(34): 25131–25140. doi: 10.1074/jbc.M702123200 17580302

[41] Lučić I, Rathinaswamy MK, Truebestein L, Hamelin DJ, Burke JE, Leonard TA. Conformational sampling of membranes by Akt controls its activation and inactivation. Proc. Natl. Acad. Sci. U. S. A. 2018; 115(17): E3940–E3949. doi: 10.1073/pnas.1716109115 29632185 PMC5924885

[42] Saxton RA, Sabatini DM. mTOR signaling in growth, metabolism, and disease. Cell. 2017; 168(6): 960–976. doi: 10.1016/j.cell.2017.02.004 28283069 PMC5394987

[43] Banerjee P, Ahmad MF, Grove JR, Kozlosky C, Price DJ, Avruch J. Molecular structure of a major insulin/mitogen-activated 70-kDa S6 protein kinase. Proc Natl Acad Sci U S A. 1990; 87(21): 8550–8554. doi: 10.1073/pnas.87.21.8550 2236064 PMC54994

[44] Shima H, Pende M, Chen Y, Fumagalli S, Thomas G, Kozma SC. Disruption of the p70(S6K)/p85(S6K) gene reveals a small mouse phenotype and a new functional S6 kinase. EMBO Journal. 1998; 17(22): 6649–6659. doi: 10.1093/emboj/17.22.6649 9822608 PMC1171010

[45] Sridharan S, Basu A. Distinct Roles of mTOR Targets S6K1 and S6K2 in Breast Cancer. International Journal of Molecular Sciences. 2020; 21(4): 1199. doi: 10.3390/ijms21041199 32054043 PMC7072743

[46] Hara K, Yonezawa K, Weng Q, Kozlowski MT, Belham C, Avruch J. Amino Acid Sufficiency and mTOR Regulate p70 S6 Kinase and eIF-4E BP1 through a Common Effector Mechanism. Journal of Biological Chemistry. 1998; 273(23): 14484–14494. doi: 10.1074/jbc.273.23.14484 9603962

[47] Gingras AC, Gygi SP, Raught B, Polakiewicz RD, Abraham RT, Hoekstra MF, et al. Regulation of 4E-BP1 phosphorylation: a novel two-step mechanism. Genes & development. 1999; 13(11): 1422–1437. doi: 10.1101/gad.13.11.1422 10364159 PMC316780

[48] Thoreen CC, Chantranupong L, Keys HR, Wang T, Gray NS, Sabatini DM. A unifying model for mTORC1-mediated regulation of mRNA translation. Nature. 2012; 485(7396): 109–113. doi: 10.1038/nature11083 22552098 PMC3347774

[49] Walsh D, Mohr I. Viral subversion of the host protein synthesis machinery. Nature Reviews Microbiology. 2011; 9(12): 860–875. doi: 10.1038/nrmicro2655 22002165 PMC7097311

[50] Lloyd RE. Translational control by viral proteinases. Virus Research. 2006; 119(1): 76–88. doi: 10.1016/j.virusres.2005.10.016 16303201 PMC7173276

[51] Willcocks MM, Carter MJ, Roberts LO. Cleavage of eukaryotic initiation factor eIF4G and inhibition of host-cell protein synthesis during feline calicivirus infection. Journal of General Virology. 2004; 85(5): 1125–1130. doi: 10.1099/vir.0.19564-0 15105529

[52] Zhan Y, Yu S, Yang S, Qiu X, Meng C, Tan L, et al. Newcastle Disease virus infection activates PI3K/Akt/mTOR and p38 MAPK/Mnk1 pathways to benefit viral mRNA translation via interaction of the viral NP protein and host eIF4E. PLoS Pathogens. 2020; 16(6): e1008610. doi: 10.1371/journal.ppat.1008610 32603377 PMC7326156

[53] Wang S, Zhao X, Wang J. Molecular cloning and characterization of the translationally controlled tumor protein from *Fenneropenaeus chinensis*. Molecular Biology Reports. 2009; 36(7): 1683–1693. doi: 10.1007/s11033-008-9369-2 18853281

[54] Li C, Hong P, Yang M, Zhao X, Wang J. FOXO regulates the expression of antimicrobial peptides and promotes phagocytosis of hemocytes in shrimp antibacterial immunity. PLoS Pathogens. 2021; 17(4): e1009479. doi: 10.1371/journal.ppat.1009479 33798239 PMC8046353

[55] Livak KJ, Schmittgen TD. Analysis of relative gene expression data using real-time quantitative PCR and the 2^−ΔΔCT^ method. Methods. 2001; 25(4): 402–408. doi: 10.1006/meth.2001.1262 11846609

[56] Elamri I, Heumüller M, Herzig LM, Stirnal E, Wachtveitl J, Schuman EM, et al. A New Photocaged Puromycin for an Efficient Labeling of Newly Translated Proteins in Living Neurons. ChemBioChem. 2018; 19(23): 2458–2464. doi: 10.1002/cbic.201800408 30311996

[57] Reichelt P, Schwarz C, Donzeau M. Single step protocol to purify recombinant proteins with low endotoxin contents. Protein Expression and Purification. 2006; 46(2): 483–488. doi: 10.1016/j.pep.2005.09.027 16290005

