# Supplementary figures and images for: White spot syndrome virus directly activates mTORC1 signaling to facilitate its replication via polymeric immunoglobulin receptor-mediated infection in shrimp
Source: PLoS Pathog. 2022 Sep 6;18(9):e1010808. doi: 10.1371/journal.ppat.1010808 (PMC9481175; doi:10.1371/journal.ppat.1010808)

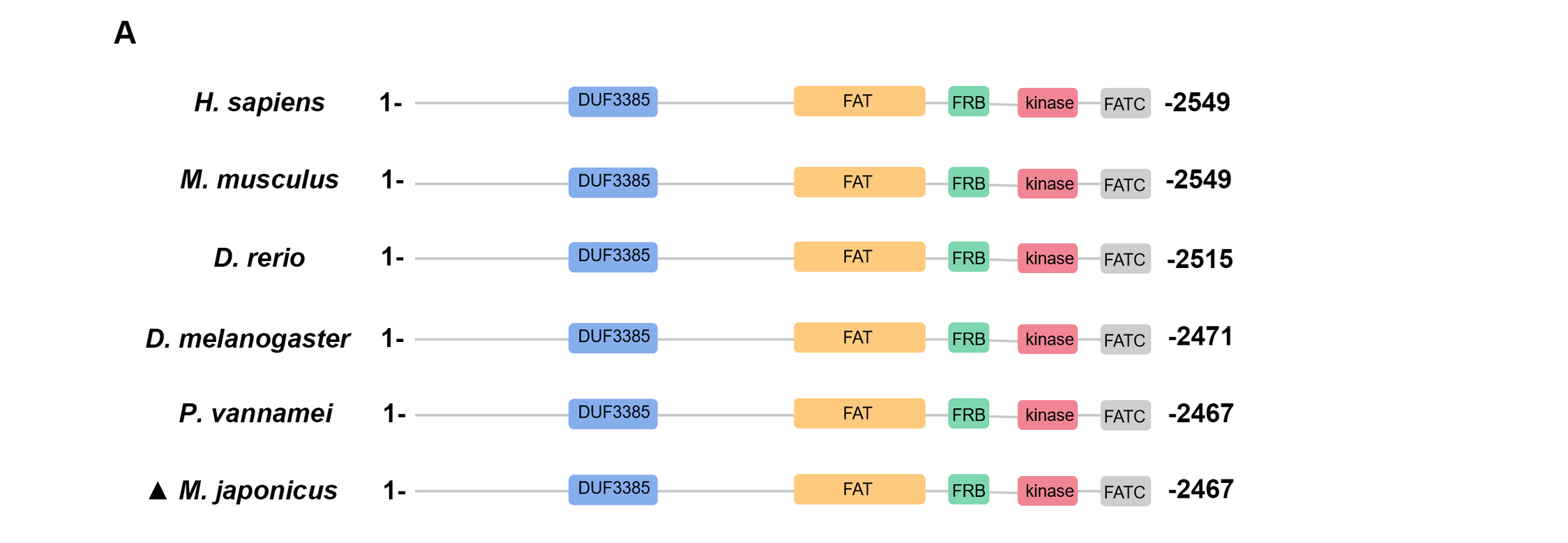

Supplement: S1 Fig — H. sapiens, Homo sapiens; M. musculus, Mus musculus; D. rerio, Danio rerio; D. melanogaster, Drosophila melanogaster; P. vannamei, Penaeus vannamei. DUF3385, the uncharacterized domain ranged from 160 to 172 amino acids in length and was identified in the phosphatidylinositol kinase-related protein kinases of mTOR: representatives of the three main groups sharing the domain FRAP, ATM, and TRRAP (FAT); rapamycin binding domain (FRB); kinase, PI3kc kinase; and FRAP, ATM, TRRAP C-terminal (FATC). (TIF) [file ppat.1010808.s001.tif]

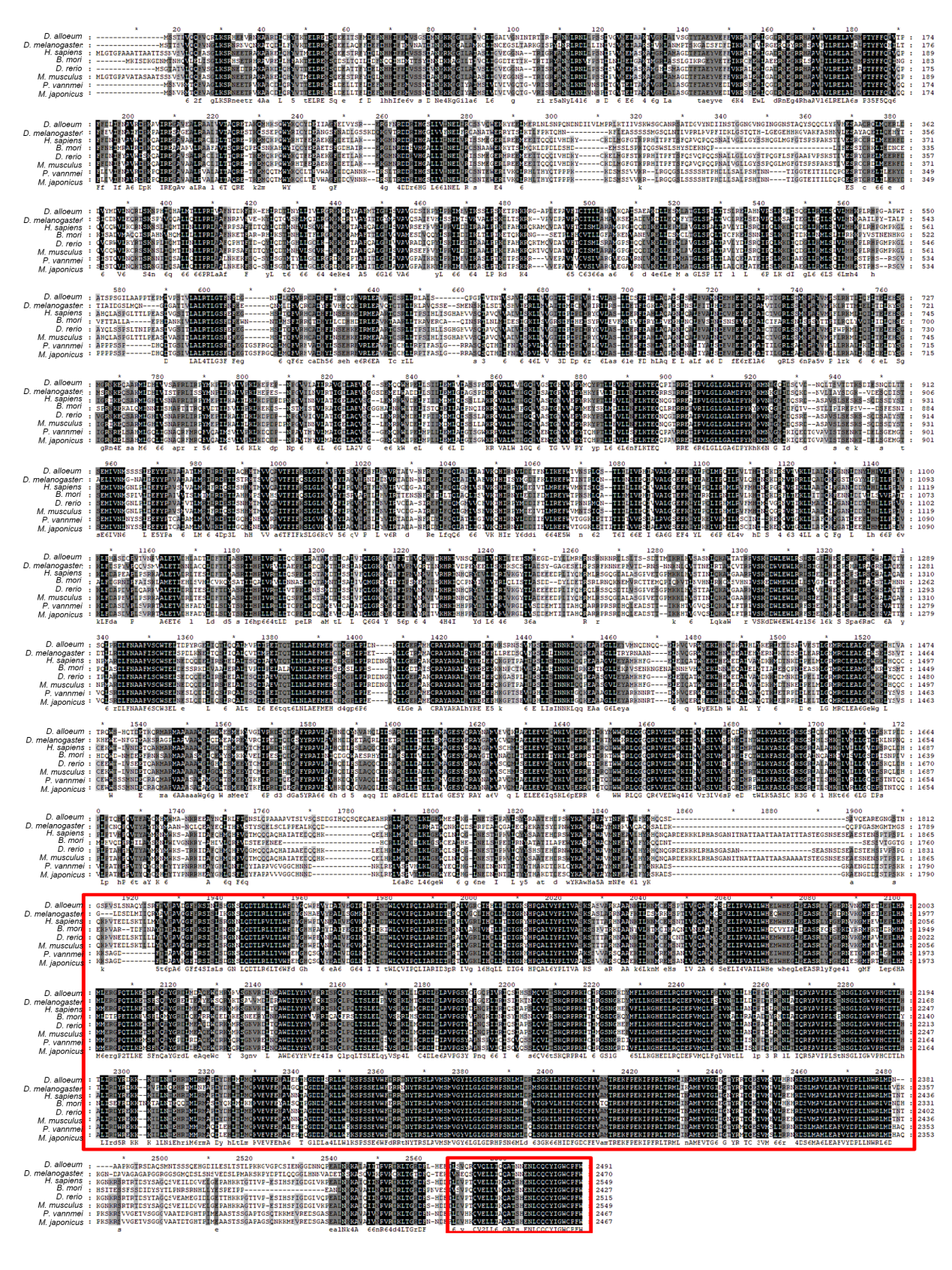

Supplement: S2 Fig — The mTOR sequences were derived from GenBank. Diachasma alloeum, XP_015118165.1; Drosophila melanogaster, NP_524891.1; Homo sapiens, NP_004949.1; Bombyx mori, NP_001171773.1; Danio rerio, ABG56082.2; Mus musculus, NP_064393.2; Penaeus vannamei, XP_027228160.1. The domains in the red box represents the kinase and FATC domains, respectively. (TIF) [file ppat.1010808.s002.tif]

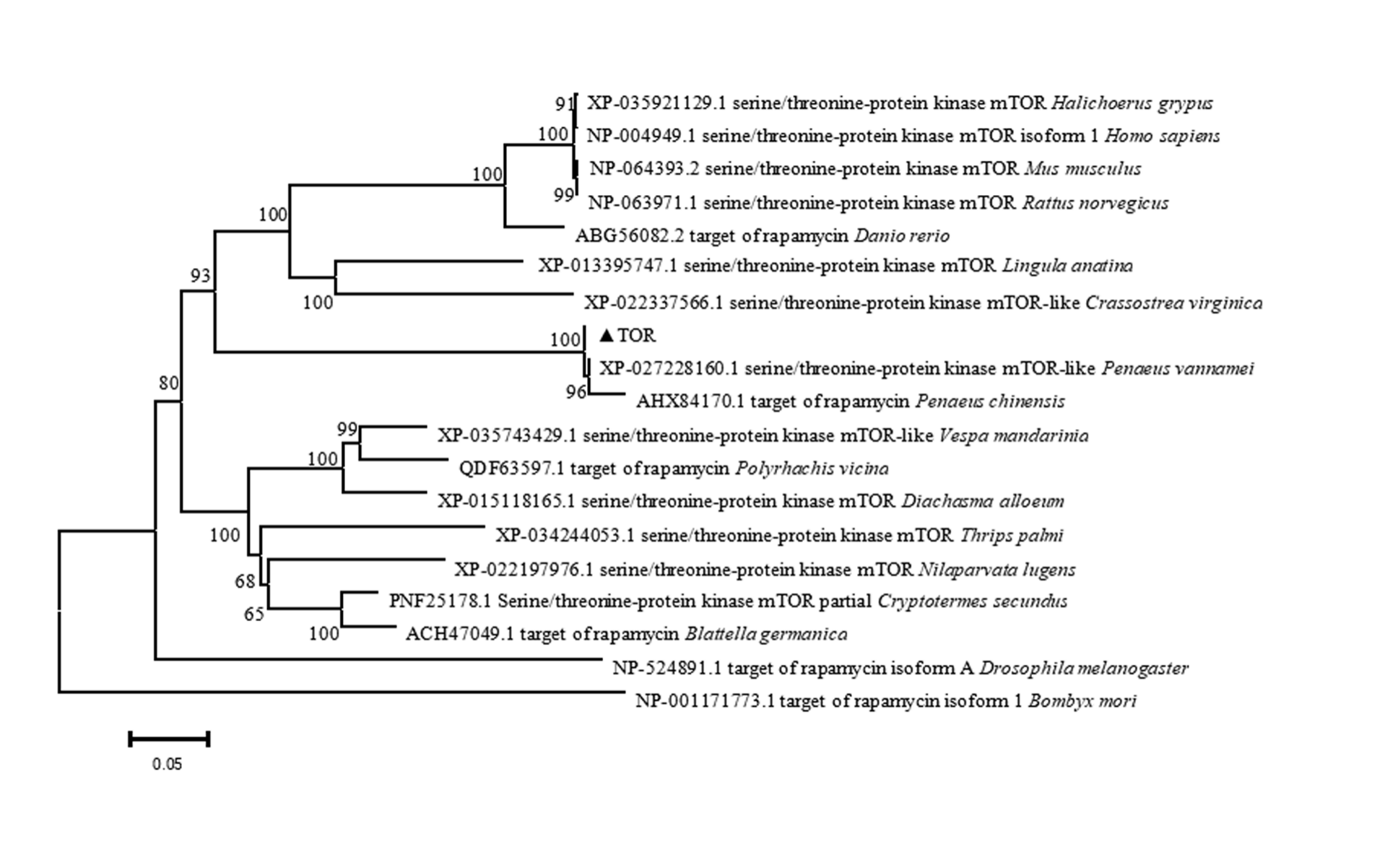

Supplement: S3 Fig — The mTOR sequences of different species were obtained from GenBank, and the NJ tree was established using MEGA 6.0. The results were repeated 1000 times by bootstrapping. The mTOR of M. japonicus is denoted by a black triangle. (TIF) [file ppat.1010808.s003.tif]

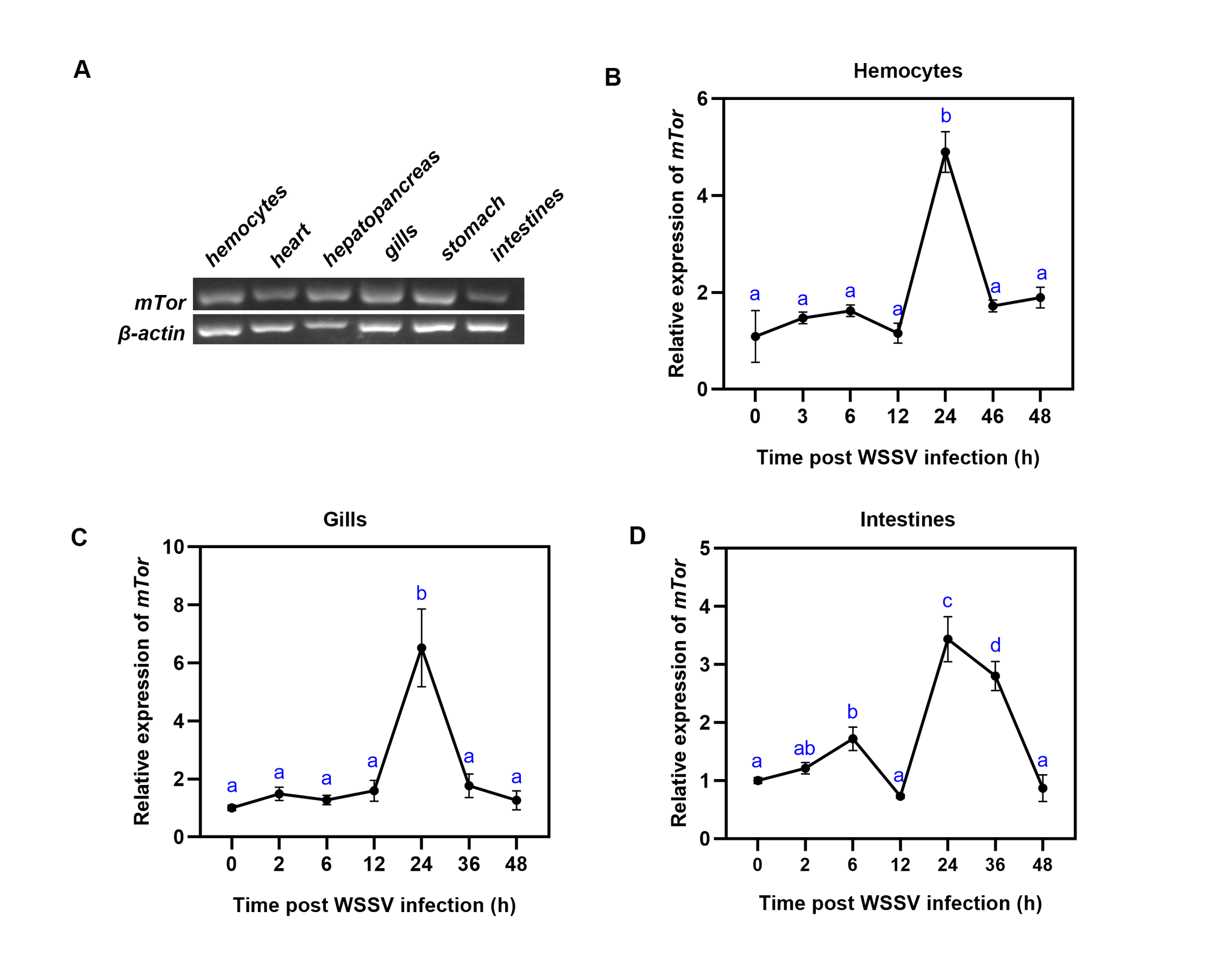

Supplement: S4 Fig — A. Tissue distribution of mTor in shrimp at the mRNA level detected using RT-PCR. B-D. Expression patterns of mTor in hemocytes (B), gills (C), and intestines (D), detected by qPCR. β-Actin was used as an internal control. Significant differences were analyzed using a Student’s t-test and P < 0.05 was accepted as a significant difference. (TIF) [file ppat.1010808.s004.tif]

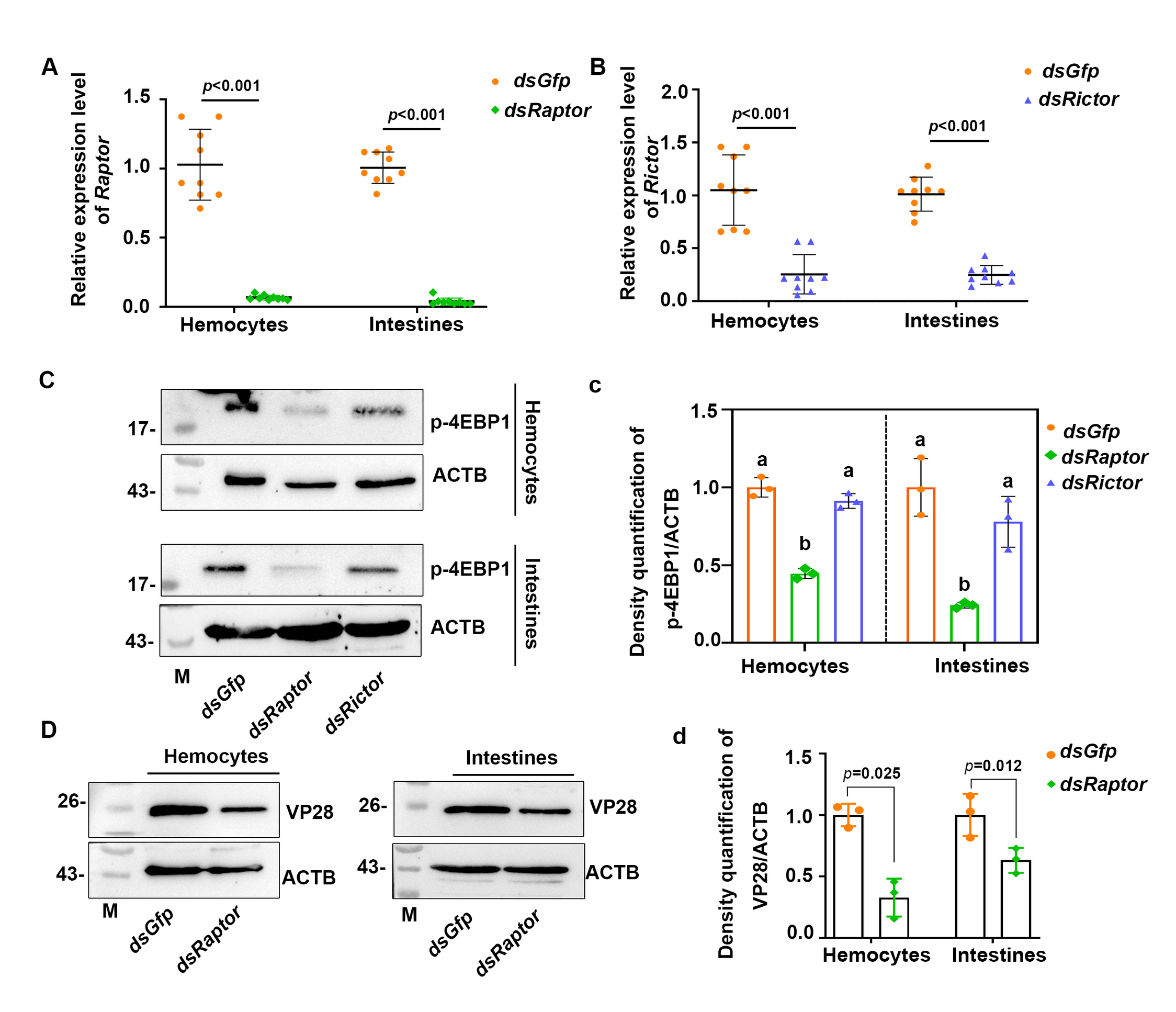

Supplement: S5 Fig — A. The efficiency of Raptor RNAi in hemocytes and intestines in shrimp as detected by qPCR. B. The efficiency of Rictor RNAi in the hemocytes and intestines detected by qPCR. C. 4EBP1 phosphorylation after the knockdown of Raptor and Rictor in the hemocytes and intestines. c. Statistical analysis of three independent experiments for panels C. D. The level of VP28 protein expression in the hemocytes and intestines of Raptor-RNAi shrimp challenged with WSSV and detected by Western blot at 36 hpi. ACTB was used as the loading control. d. Statistical analysis of three independent experiments for panel D. (TIF) [file ppat.1010808.s005.tif]

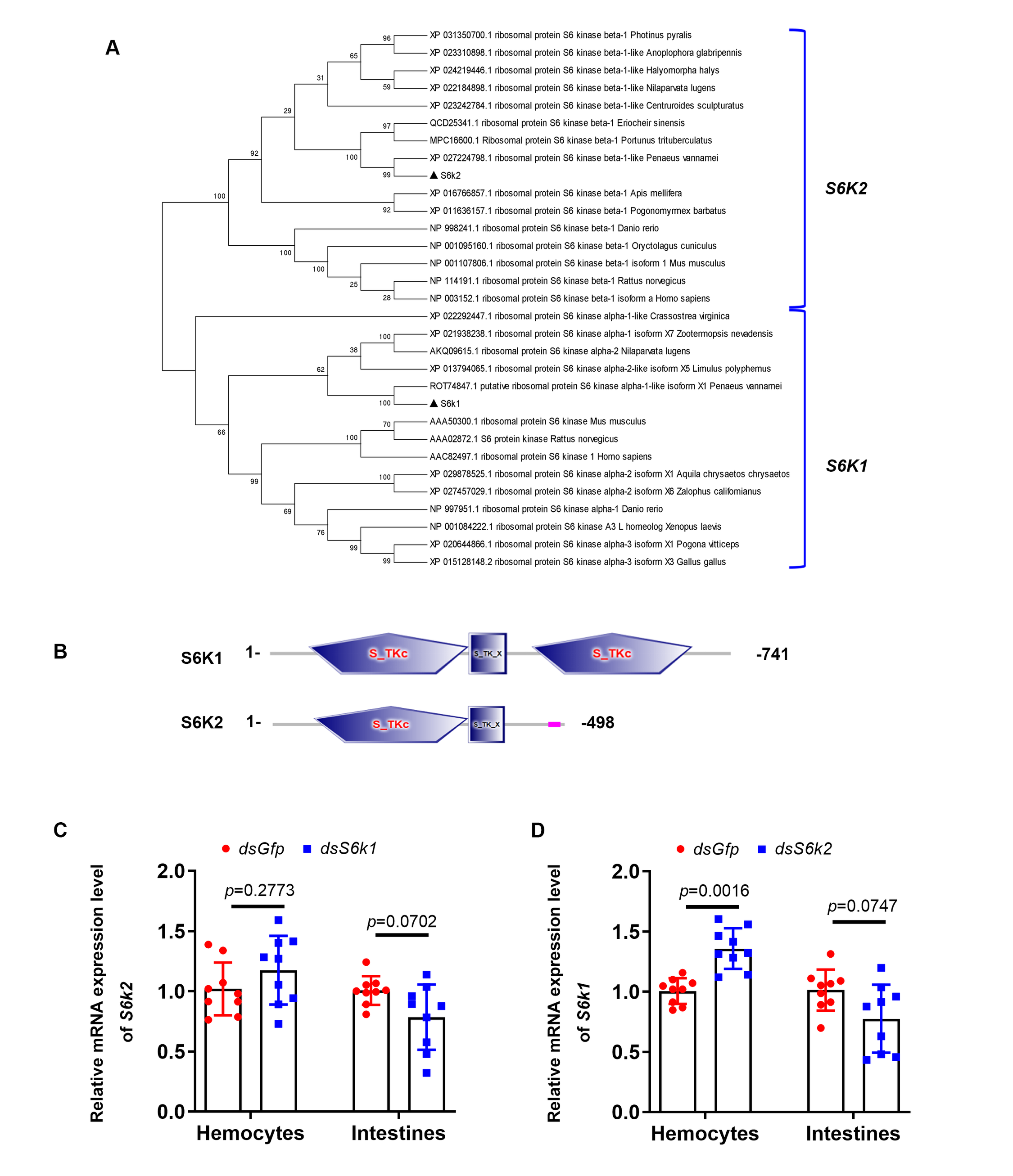

Supplement: S6 Fig — A. Phylogenetic tree of S6K1s and S6K2s from different species. S6K sequences of different species were obtained from GenBank, and an NJ tree was established using MEGA 6.0. B. Domains architecture of S6K1 and S6K2 in M. japonicus. C. The mRNA expression level of S6k2 detected by qPCR after knocking down of S6k1. D. The mRNA expression level of S6k1 detected by qPCR after knocking down of S6k2. (TIF) [file ppat.1010808.s006.tif]

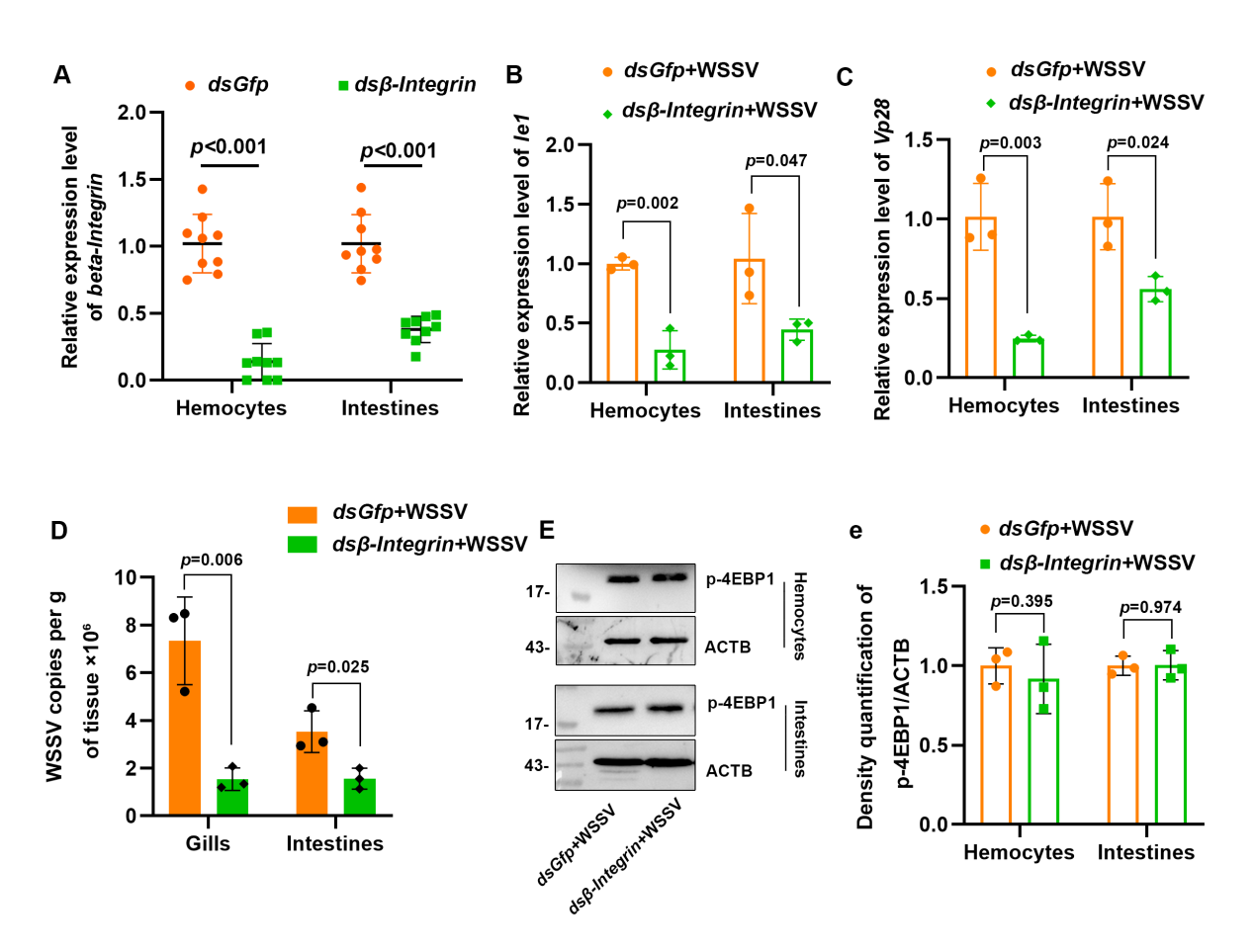

Supplement: S7 Fig — A. Efficiency of β-Integrin-RNAi in the hemocytes and intestines of shrimp analyzed by qPCR. B-C. β-Integrin knockdown, the expression of Vp28 (B) and Ie1 (C) at the transcriptional level detacted by qPCR. D. The WSSV copy number decreased significantly after β-Integrin knockdown. E. Phosphorylation of 4EBP1 was detected after knocking down of β-Integrin. e. Statistical analysis based on three independent experiments of (E). Significant differences were analyzed using a Student’s t-test, and P < 0.05 was considered to indicate a significant difference. (TIF) [file ppat.1010808.s007.tif]

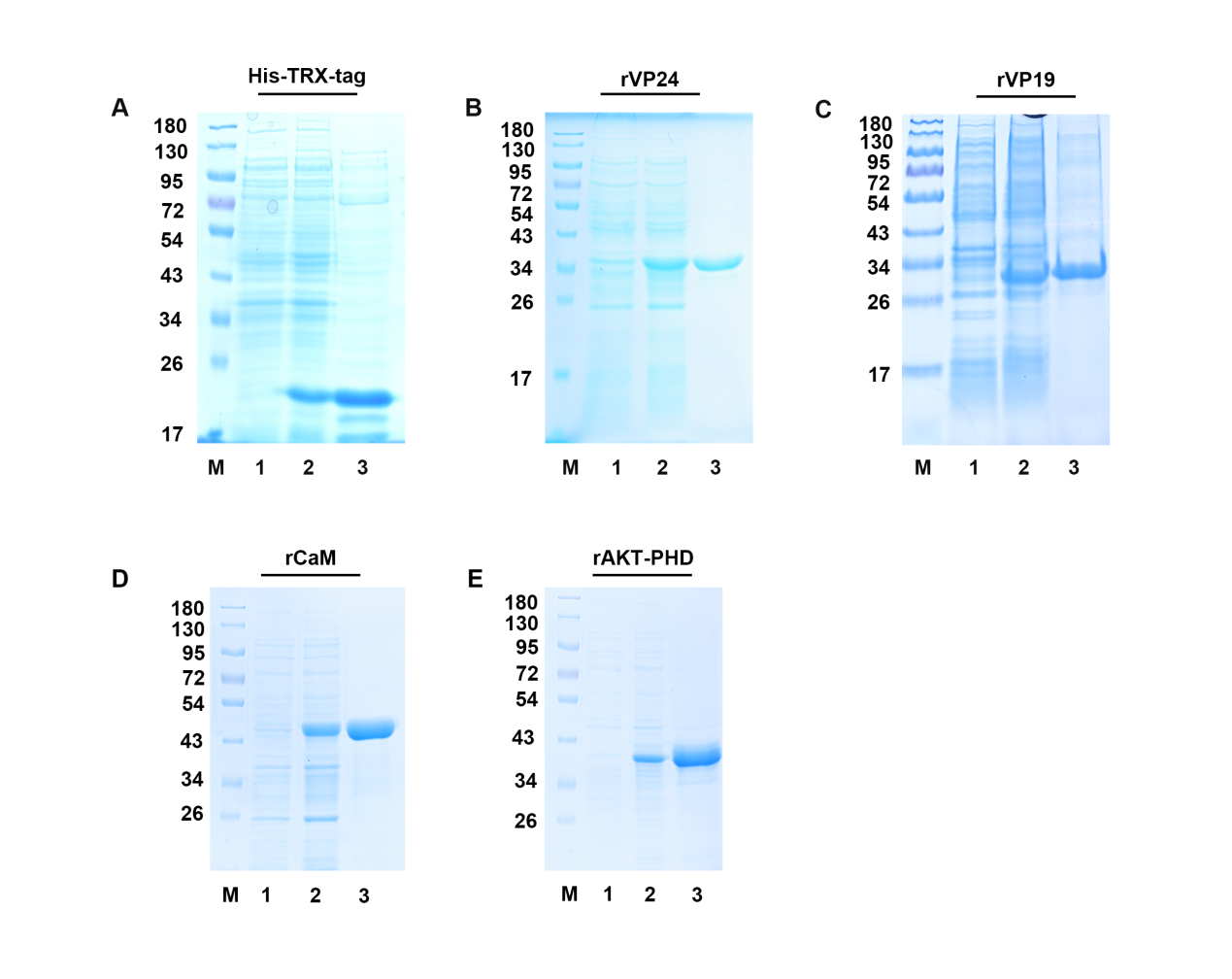

Supplement: S8 Fig — A-C. TRX-His tag (A), rVP24 (B), and rVP19 (C) expression and purification from E. coli. Lane 1, the total proteins from E. coli with pET32a (+) parental plasmid or pET32a-Vp24 or pET32a-Vp19 without IPTG induction; lane 2, total proteins from E. coli with IPTG induction; lane 3, purified recombinant proteins (TritonX-114 was used to remove endotoxins for all three of the proteins used in the in vivo injection). D-E. CaM (D) and AKT-PHD (E) expression and purification from E. coli. Lane 1, total proteins from E. coli with pGEX4T-1-CaM or pET-32a-AKT-PHD without IPTG induction; lane 2, total proteins from the E. coli with IPTG induction; lane 3, purified recombinant proteins. (TIF) [file ppat.1010808.s008.tif]

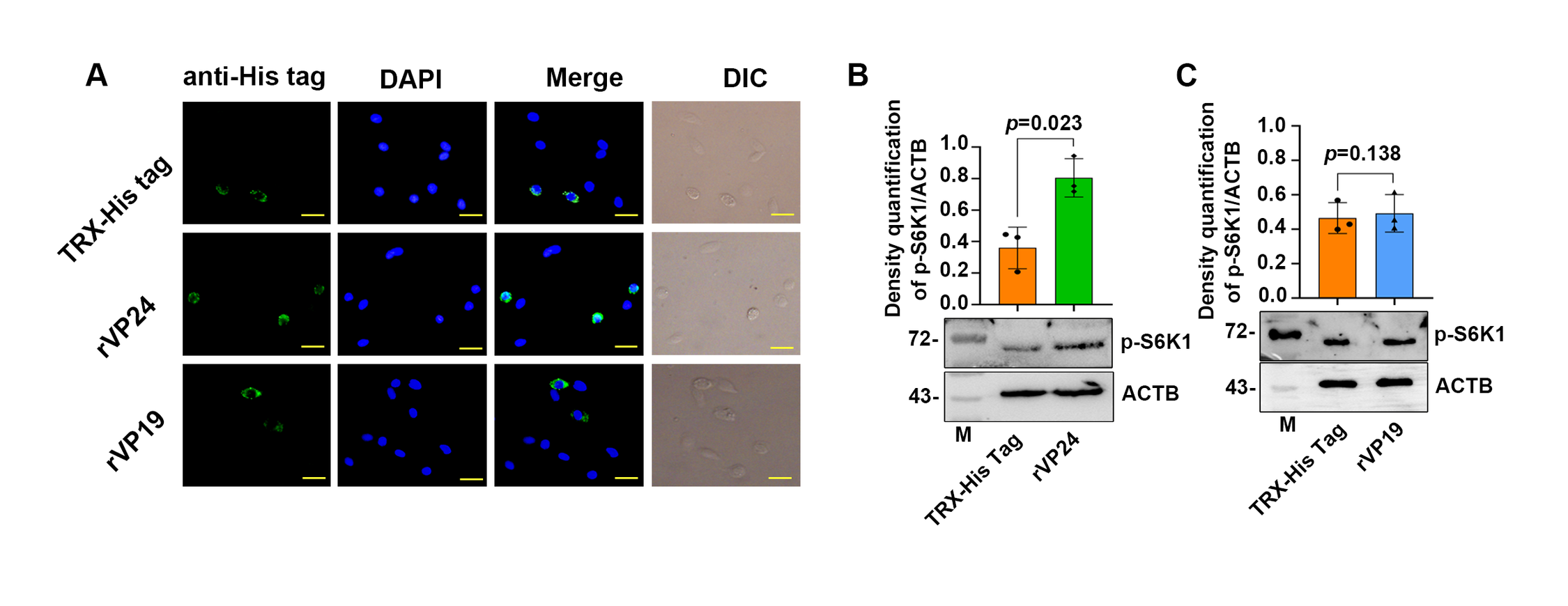

Supplement: S9 Fig — A. Immunocytochemistry was performed to detect the entry of recombinant proteins into hemocytes. DIC, differential interference construct. Scale bar = 20 μm. B-C. The phosphorylation of S6K1 in the hemocytes of shrimp injected with rVP24 (B) or rVP19 (C) compared with control group analyzed by Western blot at 24 h post proteins injection. The upper panel represents the statistical analysis of three independent experiments of the lower panel. (TIF) [file ppat.1010808.s009.tif]

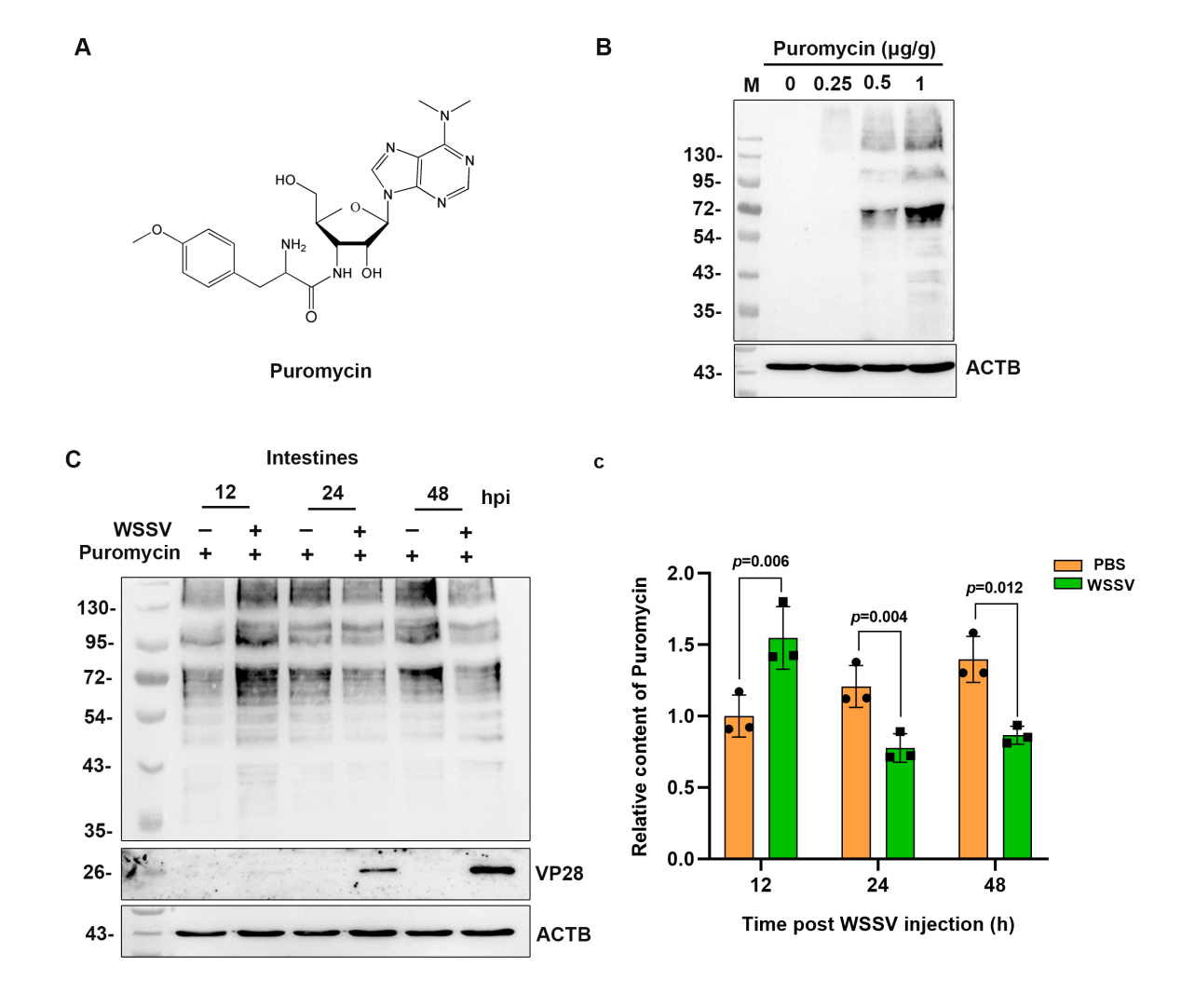

Supplement: S10 Fig — A. Structural formula of puromycin. B. The level of global protein translation labelled with different concentrations of puromycin was detected in shrimp challenged with WSSV. C. The global protein and VP28 translation were detected in the intestines of shrimp at different time points post-WSSV and PBS injection by Western blot using anti-puromycin as the primary antibody; c. Statistical analysis of panel C based on three independent experiments. Significant differences were analyzed using a Student’s t-test, and P < 0.05 was considered to indicate a significant difference. (TIF) [file ppat.1010808.s010.tif]
