# Supplementary material for: White spot syndrome virus directly activates mTORC1 signaling to facilitate its replication via polymeric immunoglobulin receptor-mediated infection in shrimp
Source: PLoS Pathog. 2022 Sep 6;18(9):e1010808. doi: 10.1371/journal.ppat.1010808 (PMC9481175; doi:10.1371/journal.ppat.1010808)
Supplement: S1 Table — Annealing temperature (°C). Amplicon size (bp). The amplification efficiency of the primers (%) (DOCX) [file ppat.1010808.s011.docx]

**S1 Table**

**Primer sequences used in this article**

| Primers | Sequence (5’-3’) | | GenBank number | T_m_^1^ (°C) | | | Size^2^  (bp) | amplification efficiency(%)^3^ | |
| --- | --- | --- | --- | --- | --- | --- | --- | --- | --- |
| RT-PCR and qPCR | | |  |  | | |  |  | |
| *mTor*-F | CTCCTCACGGTCTGGTTTG | | OK143314 | 55.9 | | | 235 | 98.03 | |
| *mTor*-R | CGGACGACTTTGCTGCTAC | |  | 56.7 | | |  |  |  |
| *S6k1-*F | CCTTTGGTGTTCTTTTGTT | | OK143316 | 47.1 | | | 211 | 96.33 | |
| *S6k1*-R | GGATGACGCTTCAGTTCTT | |  | 52.1 | | |  |  |  |
| *S6k2*-F | GCAAACTGCTCAAGAGACAAG | | OK143317 | 54.1 | | | 267 | 92.30 | |
| *S6k2*-R | CGTAGGTGAATCCCTCAAAGA | |  | 53.9 | | |  |  |  |
| *4Ebp1*-F | ATGTTTATGCTTCCCATTTGA | | OK143315 | 48.9 | | | 229 | 94.17 | |
| *4Ebp1*-R | ATTATTCTGCCCTGGTTTGTT | |  | 51.5 | | |  |  |  |
| *Cam*-F | GGACGCTGACGGTAAATG | | MH238441 | 53.7 | | | 491 | 95.30 | |
| *Cam*-R | GTGGTGGCTGCTTGATA | |  | 52.0 | | |  |  |  |
| *pIgR*-F | AAGTGTTAGCCGACAGGTTG | | MH051890 | 55.1 | | | 125 | 90.00 | |
| *pIgR*-R | CTAAGATTTGTCAGACGCACC | |  | 53.4 | | |  |  |  |
| *Raptor-*F | CAGCACTACACTCTCACCA | | OK143318 | 54.0 | | | 207 | 95.70 | |
| *Raptor-*R | TCACTTCACCAGAAACAAC | |  | 49.4 | | |  |  |  |
| *Rictor-*F | GGATGGGATGACTTGGTA | | OK143319 | 50.7 | | | 308 | 91.20 | |
| *Rictor-*R | GTGGTGAGTATGCGGTTG | |  | 53.7 | | |  |  |  |
| *Vp28- F* | AGCTCCAACACCTCCTCCTTCA | | 927064 | 60.6 | | | 162 | 101.20 | |
| *Vp28- R* | TTACTCGGTCTCAGTGCCAGA | |  | 57.8 | | |  |  |  |
| *Ie1- F* | GACTCTACAAATCTCTTTGCCA | | 926915 | 51.9 | | | 502 | 123.57 | |
| *Ie1- R* | CTACCTTTGCACCAATTGCTAG | |  | 50.8 | | |  |  |  |
| *β-actin*-F | AGTAGCCGCCCTGGTTGTAGAC | | GU645235 | 55.3 | | | 240 | 107.78 | |
| *β-actin*-R | TTCTCCATGTCGTCCCAGT | |  | 54.6 | | |  |  |  |
| *Ef-1α-*F | GGATTGCCACACCGCTCACA | | AB458256 | 61.4 | | | 223 | 98.83 | |
| *Ef-1α-*R | CACAGCCACCGTTTGCTTCAT | |  | 58.9 | | |  |  |  |
| Recombinant expression | | | | | | | | | |
| Primers | Sequence (5’-3’) | | | | GenBank number | | | T_m_^1^ (°C) | Size^2^  (bp) |
| *Cam-*Ex-F | TACTCAGGATCCATGGCGGATCAGCTGACCGAA | | | | MH238441 | | | 68.6 | 447 |
| *Cam*-Ex-R | TACTCAGTCGACCTTCGAGGTCATCATCGTGAC | | | |  |  |  | 65.2 |  |
| *Akt*-PH-F | TACTCAGGATCCAAGATTGATTACAACGCAGCA | | | | KP419289 | | | 62.1 | 489 |
| *Akt*-PH-R | TACTCACTCGAGTTTCTTCTTTGAGCTTCTC | | | |  |  |  | 59.1 |  |
| RNAi | | | | | | | | | |
| *dsmTor*-F | | GCGTAATACGACTCACTATAGGCATTGTCTACCGCATAACT | | | | OK143314 | | 64.6 | 499 |
| *dsmTor*-R | | GCGTAATACGACTCACTATAGGAGCAAGATTGAGGAGGG | | | |  |  | 65.5 |  |
| *dsS6k1*-F | | GCGTAATACGACTCACTATAGGCCTTTGGTGTTCTTTTGTT | | | | OK143316 | | 64.0 | 483 |
| *dsS6k1*-R | | GCGTAATACGACTCACTATAGGTGCTTAGAGGTGATGATTT | | | |  |  | 63.1 |  |
| *dsS6k2*-F | | GCGTAATACGACTCACTATAGGAGCTGACTGCCCTGTGAG | | | | OK143317 | | 68.1 | 444 |
| *dsS6k2*-R | | GCGTAATACGACTCACTATAGGTGTGTCCTTCTGGTTGCG | | | |  |  | 67.1 |  |
| *dsGfp*-F | | GCGTAATACGACTCACTATAGGTGGTCCCAATTCTCGTGGAAC | | | | MK371210.1 | | 68.9 | 467 |
| *dsGfp*-R | | GCGTAATACGACTCACTATAGGCTTGAAGTTGACCTTGATGCC | | | |  |  | 66.2 |  |
| *dspIgR*-F | | GCGTAATACGACTCACTATAGGCACAAGGAAGGAGTGGAGGTA | | | | MH051890 | | 67.7 | 400 |
| *dspIgR*-R | | GCGTAATACGACTCACTATAGGGGCGGTAACAATAATCAGCA | | | |  |  | 65.8 |  |
| *dsCam*-F | | GCGTAATACGACTCACTATAGGTTGTGATACATACACACA | | | | MH238441 | | 61.8 | 484 |
| *dsCam*-R | | GCGTAATACGACTCACTATAGGCGCGGTCACTTCACTTCG | | | |  |  | 68.5 |  |
| *dsRaptor-*F | | GCGTAATACGACTCACTATAGGGAGACATAATGGAACAGAAG | | | | OK143318 | | 63.2 | 414 |
| *dsRaptor-*R | | GCGTAATACGACTCACTATAGGTTGGATGAAAGAGTAACACA | | | |  |  | 62.9 |  |
| *dsRictor-*F | | GCGTAATACGACTCACTATAGGGAAGCAGATGGGAAAAATA | | | | OK143319 | | 63.3 | 471 |
| *dsRictor-*R | | GCGTAATACGACTCACTATAGGCAAGCGGAATGAATAGGAA | | | |  |  | 64.8 |  |

1. Annealing temperature (°C)
2. Amplicon size (bp)
3. The amplification efficiency of the primers (%)
